# Which Is the Best Biologic for Nasal Polyps: An Updated Network Meta‐Analysis

**DOI:** 10.1002/clt2.70114

**Published:** 2025-11-03

**Authors:** Xi Xu, Jinting Lin, Minting Luo, Qingwu Wu

**Affiliations:** ^1^ Department of Otorhinolaryngology Head and Neck Surgery The Third Affiliated Hospital of Sun Yat‐sen University Guangzhou China; ^2^ Department of Pediatrics Children’s Medical Center The Third Affiliated Hospital of Sun Yat‐sen University Guangzhou China

**Keywords:** dupilumab, meta‐analysis, nasal polyps, omalizumab, tezepelumab

## Abstract

**Background:**

Direct comparative efficacy data for biologics in chronic rhinosinusitis with nasal polyps (CRSwNP) remain limited, particularly for novel agents like tezepelumab, underscoring the need to identify optimal therapies for precision management.

**Objective:**

To rank the comparative efficacy and safety of dupilumab, tezepelumab, omalizumab, and mepolizumab versus placebo for CRSwNP using network meta‐analysis.

**Methods:**

PubMed, Embase, Web of Science, and Cochrane Library were searched from inception through April 1, 2025. Randomized controlled trials (RCTs) in adults with CRSwNP comparing biologics against placebo were eligible. PRISMA‐NMA guidelines were followed. GRADE methodology was employed for evidence certainty assessment. Two investigators independently extracted data. Fixed‐effect model network meta‐analysis was performed, with treatments ranked via surface under the cumulative ranking curve (SUCRA). The primary outcomes were Nasal Polyp Score (NPS) and safety metrics (proportion of participants with ≥ 1 adverse event). Secondary outcomes included Sino‐Nasal Outcome Test‐22 (SNOT‐22), University of Pennsylvania Smell Identification Test (UPSIT), and Nasal Congestion Score (NCS).

**Results:**

Thirteen RCTs (*n* = 2304) evaluating four biologics versus placebo were included. Compared to placebo, NPS was significantly improved by dupilumab (WMD: −2.16, 95% CI [−2.44, −1.89]), omalizumab (WMD: 1.25, 95% CI [−1.52, −0.97]), mepolizumab (WMD: 0.90, 95% CI [−1.19, −0.62]), and tezepelumab (WMD: −1.50, 95% CI [−1.81, −1.19]). Dupilumab ranked first in efficacy outcomes (NPS, SNOT‐22, UPSIT, and NCS, SUCRA ≥ 0.900, respectively). Tezepelumab ranked second in NPS (SUCRA: 0.720) and UPSIT (SUCRA: 0.749), while omalizumab ranked first in safety (SUCRA of adverse events: 0.064). GRADE assessments indicated that the certainty of the evidence was predominantly high for these key efficacy comparisons.

**Conclusions:**

Dupilumab demonstrated the highest efficacy and safety profile. Tezepelumab showed comparable efficacy in NPS with omalizumab.

## 1 Introduction

Chronic rhinosinusitis (CRS) is a prevalent inflammatory disease affecting the nasal mucosa and paranasal sinuses, with an incidence from 5.5% to 28% in the general population [1, 2]. Approximately 25%–30% of CRS patients suffer from chronic rhinosinusitis with nasal polyps (CRSwNP) [3]. CRSwNP is a severe CRS subtype characterized by the presence of nasal polyps and often causes clinical symptoms such as nasal obstruction, rhinorrhea, and anosmia, significantly impairing patients’ quality of life [4, 5]. Current management strategies for CRSwNP include intranasal corticosteroid (INCS), systemic corticosteroid and surgery, yet face ongoing high recurrence rates.

The pathophysiology of CRSwNP appears to be predominantly mediated by the type 2 inflammatory pathway. Previous studies indicate that biologics targeting the Th2 inflammatory pathway—such as anti‐IgE omalizumab [6, 7], anti‐IL‐5 mepolizumab [8, 9, 10], and anti‐IL‐4Rα dupilumab [11, 12]—exhibit significant efficacy for CRSwNP patients. The patients meet the specific clinical criteria: inadequate response to ≥ 4 weeks of INCS therapy or high baseline disease severity (Nasal Polyp Score [NPS] ≥ 5) and/or severe anosmia (University of Pennsylvania Smell Identification Test [UPSIT] ≤ 15) [13]. As well as further indicated for patients with comorbid asthma/atopic conditions and serve as surgical‐sparing alternatives [14, 15, 16].

In recent years, research on biologics has advanced rapidly, particularly with tezepelumab—a novel agent targeting thymic stromal lymphopoietin (TSLP), an upstream regulator of Th2 inflammation. Tezepelumab has shown remarkable efficacy in allergic inflammation [17], especially for severe uncontrolled asthma [18]. As an epithelial‐derived key alarmin, TSLP drives the Th2 inflammatory cascade by activating dendritic cells, mast cells, and type 2 innate lymphoid cells (ILC2s), thereby contributing to shared pathophysiological mechanisms in severe asthma and CRSwNP [19, 20, 21]. Preclinical studies suggest that TSLP inhibitors may exhibit broader‐spectrum anti‐inflammatory effects compared to conventional downstream Th2‐targeting biologics [22], potentially offering unique benefits for patients with mixed inflammatory phenotypes or inadequate responses to existing therapies. However, the efficacy profile and optimal target population of tezepelumab in CRSwNP remain unclear, as prior network meta‐analyses have not included this agent. Notably, a multicenter phase 3 trial demonstrated that tezepelumab significantly improved endoscopic scores (*p* < 0.001) and nasal congestion symptoms (*p* = 0.002) in CRSwNP patients [23], providing high‐level evidence to support its safety and efficacy beyond preliminary findings.

In conclusion, the comparative efficacy of established type 2 inflammatory biologics (omalizumab, dupilumab, and mepolizumab) versus the novel TSLP inhibitor tezepelumab remains uncertain. Based on our previous studies [14, 15, 16], we aim to update randomized controlled trials (RCTs) and integrate tezepelumab into a network meta analysis (NMA) of CRSwNP biologics. By synthesizing direct and indirect comparisons, we evaluated critical outcomes including polyp size reduction, quality‐of‐life improvement, olfactory recovery, and adverse event profiles. This comprehensive approach elucidates the relative therapeutic advantages of each biologic in CRSwNP management. Furthermore, we provide objective intervention rankings based on surface under the cumulative ranking curve (SUCRA) values derived from the NMA. These findings aim to inform evidence‐based, individualized treatment strategies in clinical practice.

## 2 Methods

We performed this NMA in strict accordance with established methodological standards, including the Cochrane Handbook for Systematic Reviews, GRADE evaluation guidelines [24], and PRISMA‐NMA reporting guidelines [25]. The study protocol was registered in the PROSPERO international register of systematic reviews (No. CRD420251003754), which was updated retrospectively after analyses were complete.

### 2.1 Main Outcomes

The primary outcomes were NPS and safety metrics (proportion of participants with ≥ 1 adverse event). Secondary outcomes included Sino‐Nasal Outcome Test‐22 (SNOT‐22), UPSIT, and Nasal Congestion Score (NCS).

### 2.2 Search Strategy

This study was conducted in strict accordance with PRISMA guidelines. Two independent reviewers (Xi Xu and Minting Luo) performed a comprehensive literature search of PubMed, Embase, Cochrane Library, and Web of Science databases from inception through April 1st, 2025. The search strategy was developed using the PICOS framework, incorporating key terms including “nasal polyps,” “sinusitis,” “antibodies, monoclonal,” “omalizumab,” and subject headings for “tezepelumab,” along with additional supplementary terms (complete search strategy available in online Supporting Information S1: eTable 1).

Following initial screening for potential relevance, full‐text articles were independently assessed for eligibility. To ensure thoroughness, the reference lists of all included studies were manually reviewed by the same reviewers. Any discrepancies during the screening process were resolved through discussion with a third reviewer (Jinting Lin), thereby maintaining methodological rigor throughout the study selection process.

### 2.3 Article Selection Criteria

Literature inclusion criteria: (a) Study population: adult patients with CRSwNP (age ≥ 18 years); (b) Intervention: biologics including dupilumab, omalizumab, mepolizumab, and tezepelumab, in combination with nasal glucocorticosteroids or monotherapy, with a course of administration of at least 8 weeks, and a followup duration of at least 8 weeks; (c) Control group: placebo or other biologic; (d) Study type: RCTs (e) Data requirements: provide complete reporting of outcome data (f) Study language: written and published in English.

Literature exclusion criteria: (a) non‐randomized studies such as retrospective analyses, case reports, animal experiments; (b) single‐arm design studies; (c) letters to the editor, reviews, or conference abstracts; (d) studies that were duplicative or had opaque sources of data; (e) use of other biologics in the intervention group than the four biologics described in this article or the combination of nasal polyp surgery (ESS) (f) failure to provide detailed abstracts or full‐text articles.

### 2.4 Data Extraction and Synthesis

Two researchers with extensive experience in rhinology completed data extraction using a standardized form, and used EndNote software for literature management and de‐duplication, which included basic information about the study (authors, year of publication, sample size, and country), patient characteristics (age, gender, CRSwNP diagnostic criteria and comorbidities), details of the intervention (type of biologics, dosage, duration), outcome indicators (NPS, UPSIT, NCS, SNOT‐22, AEs) in Table 1. The duration of follow‐up for the assessed outcomes varied across the included studies. To capture the most comprehensive treatment effect, we extracted outcome data at the longest available follow‐up time point reported for each study. For the SINUS 52 [12] and SYNAPSE [9] trials, both 52‐week studies, the outcomes analyzed reflect data collected at 52 weeks. Continuous variables were documented as means with standard deviations, while categorical variables were presented as frequencies and percentages. Data integrity was ensured through cross‐validation with clinical trial registry information. For studies that did not report the standard deviation (SD) of change values, Cochrane Handbook guidelines and data from similar studies were relied upon.

**TABLE 1:** Characteristics of included studies.

Study, year	Country	Subject	Population	Comorbidity		Intervention	Dosage	Placebo	Treatment length	Follow‐up length	Mean age years (SD)	Male *n* (%)	% with prior NP surgery
				% with asthma	% with AERD								
Bachert (SINUS 24) [12]	Belgium	276	CRSwNP	58	30	Dupilumab	300 mg sc every 2 weeks	Injection same dose and frequency	24 weeks	24 weeks	50.5 (13.4)	158 (57)	72
Bachert (SINUS 52) [12]	Belgium	448	CRSwNP	60	27	Dupilumab	300 mg sc every 2 weeks	Injection same dose and frequency	52 weeks	52 weeks	52.0 (12.4)	278 (62)	58
Bachert [11]	Belgium	60	CRSwNP	58	32	Dupilumab	300 mg sc weekly	Injection same dose and frequency	16 weeks	16 weeks	48.4 (9.4)	34 (57)	58.3
LIBERTY QUEST [26]	Multiple countries	193	CRSwNP	100	35	Dupilumab	300 mg sc every 2 weeks	Injection same dose and frequency	52 weeks	52 weeks	51.6 (13.0)	70 (36.3)	^a^
LIBERTY^b^ VENTURE [27]	Multiple countries	72	CRSwNP	100	^a^	Dupilumab	300 mg sc every 2 weeks	Injection same dose and frequency	24 weeks	24 weeks	^a^	^a^	^a^
Han (SYNAPSE) [9]	USA	407	CRSwNP	71	27	Mepolizumab	100 mg sc every 4 weeks	Injection same dose and frequency	52 weeks	52 weeks	48.8 (13.1)	265 (65)	100
Bachert [8]	Belgium	107	CRSwNP	78	NR	Mepolizumab	750 mg iv every 4 weeks	Injection same dose and frequency	24 weeks	25 weeks	50.5 (10.5)	76 (71)	100
Gevaert [10]	Belgium	30	CRSwNP	43	17	Mepolizumab	750 mg iv × 2 doses 28 days apart	Injection same dose and frequency	8 weeks	8 weeks	48.7 (9.8)	22 (73)	77
Gevaert (POLYP 1) [6]	Belgium	138	CRSwNP	54	20	Omalizumab	75–600 mg sc every 2–4 weeks	Injection same dose and frequency	24 weeks	24 weeks	51 (13.2)	88 (64)	57
Gevaert (POLYP 2) [6]	Belgium	127	CRSwNP	61	35	Omalizumab	75–600 mg sc every 2–4 weeks	Injection same dose and frequency	24 weeks	24 weeks	50.1 (11.9)	83 (65)	62
Gevaert [28]	Belgium	24	CRSwNP	100	52	Omalizumab	75–375 mg sc every 2–4 weeks	Injection same dose and frequency	16 weeks	16 weeks	49 (10)	17 (70)	83
Pinto [7]	USA	14	CRSwNP	57	NR	Omalizumab	0.016 mg/kg/IgE(IU/mL) sc every 2–4 weeks	Injection same dose and frequency	24 weeks	24 weeks	45.9 (9.5)	10 (71)	100
Lipworth [23]	UK	408	CRSwNP	60	17	Tezepelumab	210 mg sc every 4 weeks	Injection same dose and frequency	52 weeks	12 week/24 week	49.7 (13.6)	266 (65.2)	71.3

^a^ Baseline demographics not reported separately for the CRSwNP subgroup.

^b^ Included in NMA but excluded from the pooled baseline characteristics.

To evaluate the clinical significance of treatment effects, we applied minimal clinically important differences (MCIDs) for all critical outcomes. For NPS, the MCID was defined as *a* ≥ 1‐point [6, 8, 9, 10, 11, 23] or 2‐point [6, 9, 11, 12, 23] improvement based on consensus thresholds in trials; For NCS, the MCID was set at *a* ≥ 1‐point reduction [6, 23]; For SNOT‐22, the MCID was 8.9 [9]; No validated MCID exists for UPSIT smell testing (Supporting Information S1: eTable 2).

### 2.5 Assessment of Risk of Bias

The quality of the included RCTs was evaluated using the Cochrane Risk of Bias Assessment Tool, and the certainty of the evidence was assessed using the Grades of Recommendation Assessment, Development and Evaluation (GRADE) criteria. Supporting Information S1: eTable 3 summarizes the outcomes; the certainty of evidence for all direct comparisons was graded as moderate to high [29].To detect potential publication bias, we conducted funnel plot assessments (Supporting Information S1: eFigure 2). Two reviewers conducted an independent risk assessment based on the following six core indicators, including the randomization process, blinding of interventions, blinding of outcome assessment, completeness of outcome data, selective reporting, and other potential sources of bias. Outcomes were categorized by criteria into three categories: low risk, high risk, or some concerns. All disagreements were negotiated through two reviewers to reach consensus, resulting in a consistent risk of bias determination report, and the overall quality assessment results are presented in a visualization chart.

### 2.6 Statistical Analysis

A stratified strategy was used for the statistical analysis of this study: pairwise meta‐analysis were completed via Review Manager 5.4, and dichotomous outcomes were calculated using the Mantel–Haenszel method to calculate risk ratios (RR) and 95% confidence intervals (CI); data on consecutive endpoints that were included all originated from the same scale, and therefore integration of the mean changes from baseline to follow‐up and report weighted mean difference (WMD). NMA was implemented by R 4.3.1 software to construct nonparametric mixed‐effects models to assess indirect comparisons of multiple interventions, and a frequentist framework was used to validate the assumption of transmissibility. The significance threshold for all analyses was set at *p* < 0.05, and effect size ordering was realized by SUCRA. Due to the small number of included RCTs (*n* = 13), if a random effects model was used, it may lead to distortion in weight allocation due to the estimation error of inter‐study heterogeneity (τ^2^), which in turn increases the risk of bias. Therefore, a fixed‐effects model was selected for the combined analysis in this study. Nevertheless, we acknowledge the inherent limitations of the fixed‐effects model. As additional high‐quality studies emerge in this field, it would be prudent to re‐examine the extent of heterogeneity and adopt a random‐effects framework to more comprehensively account for between‐study variability.

## 3 Results

### 3.1 Study Selection and Characteristics

**FIGURE 1:**
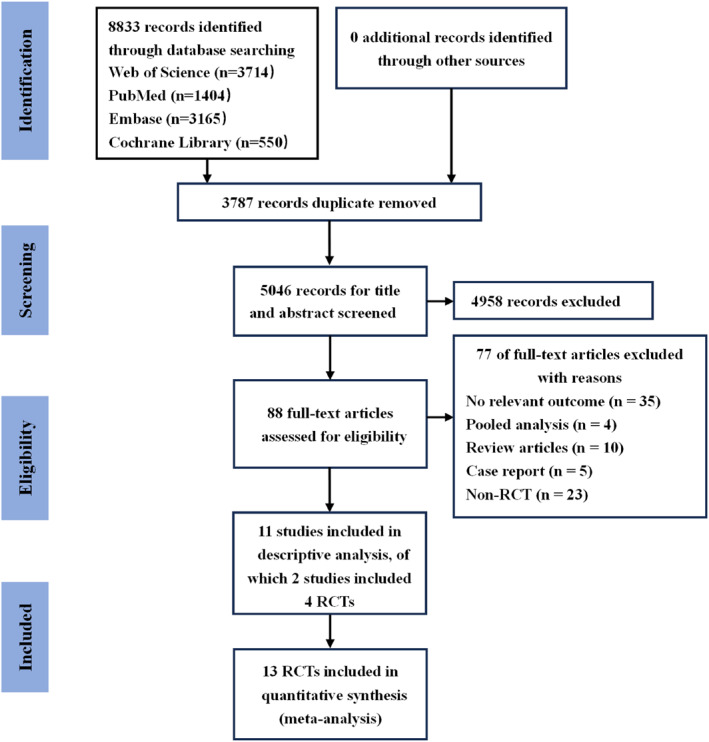
PRISMA flow diagram of the literature search.

**FIGURE 2:**
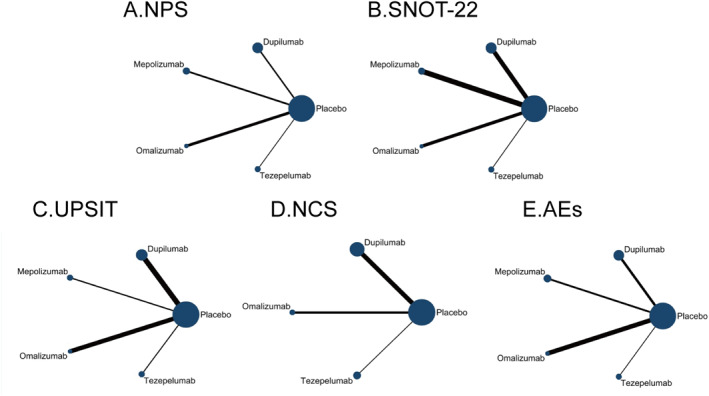
Evidence network of eligible comparisons for an network meta‐analysis: (A) NPS (nasal polyp score); (B) SNOT‐22 (Sino‐Nasal Outcome Test‐22); (C) UPSIT (University of Pennsylvania Smell Identification Test); (D) NCS (nasal congestion score); (E) AEs (adverse events). The width of the lines represents the standard deviation of comparisons, and the size of each node corresponds to the number of participants.

In this study, a total of 8833 documents were obtained through a systematic search (Figure 1), after deduplication and screening of titles and abstracts, 8745 documents that did not meet the inclusion criteria were excluded, and full text assessment was performed on the remaining 88 documents to further exclude 77 (including 35 that did not report the relevant endpoints, 23 that were not RCTs), and finally 13 RCTs were included for NMA, and the study characteristics were showed in Table 1. A total of 2304 patients with CRSwNP were included in the study. Since the LIBERTY VENTURE trial did not report baseline characteristics for the CRSwNP subgroup, it was included in the network meta‐analysis but excluded from the pooled summaries of demographic and clinical baseline characteristics. Baseline characteristics were calculated from the available data across the included studies. The mean age was 50.3 years old, 61.2% were male, 64% had a history of surgery for nasal polyps, 65.3% had comorbid asthma, and 26% had aspirin‐exacerbated respiratory disease (AERD). The included studies were all randomized controlled trials, the majority of which were two‐armed. One three‐armed trial (SINUS‐52 [12], comparing two active interventions against a common placebo control) was also included; the direct comparison evidence network diagram is shown in Figure 2.

Two large RCTs (MUSCA [30], NCT01066104) were excluded from efficacy analyses despite including eligible CRSwNP patients. Their exclusion resulted from unreported critical CRSwNP‐specific endpoints, as the absence of quantifiable polyp outcome data precluded assessment of disease‐specific treatment effects.

### 3.2 Risk of Bias and Grade Assessment

A total of 13 RCTs were included in this study, and the methodological quality was systematically evaluated by the Cochrane Risk of Bias Assessment Tool (Supporting Information S1: eFigure 1). The results showed that six studies presented a low risk of bias in random sequence generation, allocation concealment, and double‐blind implementation; five studies were judged to be at uncertain risk in the domain of blinding of outcome assessment, and one study was at some concerns for incomplete outcome data. We applied the GRADE framework to evaluate evidence certainty, revealing predominantly high‐quality evidence across included studies. GRADE evidence certainty assessment demonstrated that high certainty (predominant across studies) indicates effect estimates are robust to new evidence; moderate certainty signifies estimates may undergo meaningful modification with future research; low certainty reflects a high probability of substantial estimate changes; while very low certainty denotes extreme uncertainty in validity (Supporting Information S1: eTable 3).

### 3.3 Pair‐Wise Meta‐Analysis

**FIGURE 3:**
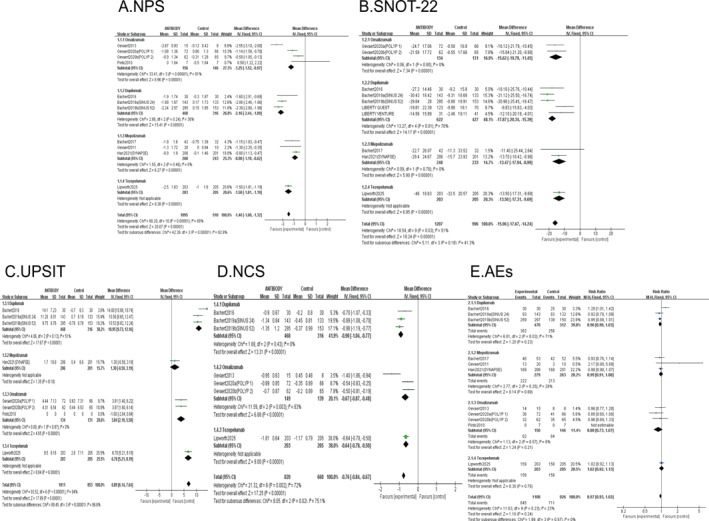
Pair‐wise meta‐analysis comparing: (A) NPS (nasal polyp score); (B) SNOT‐22 (Sino‐Nasal Outcome Test‐22); (C) UPSIT (University of Pennsylvania Smell Identification Test); (D) NCS (nasal congestion score); (E) AEs (adverse events).

The outcomes of the pairwise meta‐analysis are presented in Figure 3 and Supporting Information S1: eTable 4, quantified as WMD or RR accompanied by 95% CI.

NPS: In this study, omalizumab, dupilumab, mepolizumab, and the addition of tezepelumab all demonstrated statistically significant superiority over placebo. (WMD: −1.25, 95% CI [−1.52, −0.97]; −2.16, 95% CI [−2.44, −1.89]; −0.90, 95% CI [−1.19, −0.62]; −1.50. 95% CI [−1.81, −1.19], all *p* < 0.00001). Heterogeneity analysis showed high heterogeneity for the omalizumab comparison (*I* ^2^ = 91%), while the other three groups had negligible heterogeneity (*I* ^2^ = 0%).

SNOT‐22: Omalizumab (WMD: −15.62, 95% CI [−19.79, −11.45], *p* < 0.00001) and dupilumab (WMD: −17.87, 95% CI [−20.34, −15.39], *p* < 0.00001) significantly improved SNOT‐22 scores. Mepolizumab also showed significant efficacy after inclusion in the new study (WMD: −13.47, 95% CI [−17.94, −8.99], *p* < 0.00001). The addition of tezepelumab also performed well (WMD: −13.50, 95% CI [−17.31, −9.69], *p* < 0.00001). Heterogeneity was 0% for all comparisons.

UPSIT: Dupilumab (WMD: 10.95, 95% CI [9.73, 12.16], *p* < 0.00001), tezepelumab (WMD: 6.70, 95% CI [5.21, 8.19], *p* < 0.00001), and omalizumab (WMD: 3.84, 95% CI [2.19, 5.50], *p* < 0.00001) were significantly superior to placebo. Heterogeneity was moderate in the dupilumab group (*I* ^2^ = 51%), suggesting possible methodological differences between studies. Mepolizumab (WMD: 1.30, 95% CI [−0.59, 3.19], *p* = 0.18). Olfactory function was evaluated using the Sniffin’ Sticks Screening‐12 test rather than the UPSIT in the mepolizumab trial [8], precluding data integration in the meta‐analysis.

NCS: Omalizumab (WMD: −0.67, 95% CI [−0.87, −0.48], *p* < 0.00001), dupilumab (WMD: −0.90, 95% CI [−1.04, −0.77], *p* < 0.00001), and tezepelumab (WMD: −0.64, 95% CI [−0.78, −0.50], *p* < 0.00001) both significantly relieved nasal congestion. Heterogeneity was higher in the omalizumab group (*I* ^2^ = 83%). The NCS results for mepolizumab were not included in the analysis due to the use of a visual analog scale to assess nasal congestion [8].

AEs: Omalizumab (RR: 0.88, 95% CI [0.73, 1.07], *p* = 0.21), dupilumab (RR: 0.96, 95% CI [0.90, 1.03], *p* = 0.23), mepolizumab (RR: 0.99, 95% CI [0.91, 1.08], *p* = 0.84), and tezepelumab (RR: 1.02, 95% CI [0.92, 1.13], *p* = 0.76) were not significantly different from the placebo group in terms of risk of adverse events (AEs). Heterogeneity was higher for the dupilumab comparison (*I* ^2^ = 71%).

### 3.4 Network Meta‐Analysis

All evaluated biologics demonstrated significant efficacy in reducing NPS compared to placebo, with dupilumab exhibiting the greatest treatment effect (WMD: −2.16, 95% CI [−2.44, −1.89]), significantly better than omalizumab (WMD: −0.92, 95% CI [−1.31, −0.53]), mepolizumab (WMD: −1.26, 95% CI [−1.65, −0.86]), and tezepelumab (WMD: −0.67, 95% CI [−1.08, −0.25]); tezepelumab had the second‐best improvement (WMD: −1.50, 95% CI [−1.81, −1.19]), which was not statistically different from omalizumab (WMD: 0.25, 95% CI [−0.16, 0.67]), but was significantly superior to mepolizumab (WMD: 0.59, 95% CI [0.17, 1.01]). There was no statistical difference between omalizumab and mepolizumab (WMD: 0.34, 95% CI [−0.05, 0.73]).

Analysis of the SNOT‐22 scores indicated that all biologics demonstrated significant efficacy compared to placebo, with no statistically significant differences detected between them. The greatest numerical improvement was observed with dupilumab (WMD: −17.86, 95% CI [−20.33, −15.39]), followed by omalizumab (WMD: −15.63, 95% CI [−19.83, −11.48]), mepolizumab (WMD: −13.47, 95% CI [−17.97, −8.99]) and tezepelumab (WMD: −13.51, 95% CI [−17.32, −9.72]) had a similar effect.

Assessment of UPSIT demonstrated that dupilumab was significantly more efficacious than the other three monoclonal antibodies (omalizumab: WMD: 6.63, 95% CI [4.57, 8.69]; mepolizumab: WMD: 9.19, 95% CI [6.93, 11.45]; tezepelumab: WMD: 3.78, 95% CI [1.85, 5.71]), followed by tezepelumab, which showed statistically non‐significant superiority over omalizumab (WMD: −2.85, 95% CI [−5.08, −0.63]) and mepolizumab (WMD: −5.42, 95% CI [−7.73, −2.99]). Additionally, omalizumab was significantly more efficacious than mepolizumab. (WMD: −2.56, 95% CI [−5.06, −0.05]).

In terms of NCS relief, dupliumab (WMD: −0.90, 95% CI [−1.04, −0.77]), omalizumab (WMD: −0.67, 95% CI [−0.86, −0.48]), and tezepelumab (WMD: −0.65, 95% CI [−0.78, −0.50]) were significantly better than Placebo. There was a statistically significant difference between tezepelumab and dupilumab (WMD: −0.26, 95% CI [−0.45, −0.069]), and no statistically significant difference with omalizumab (WMD: −0.03, 95% CI [−0.27, 0.20]).

AEs showed no significant difference between the biologics and placebo (Supporting Information S1: eTable 5).

### 3.5 Ranking of the Biologics

**FIGURE 4:**
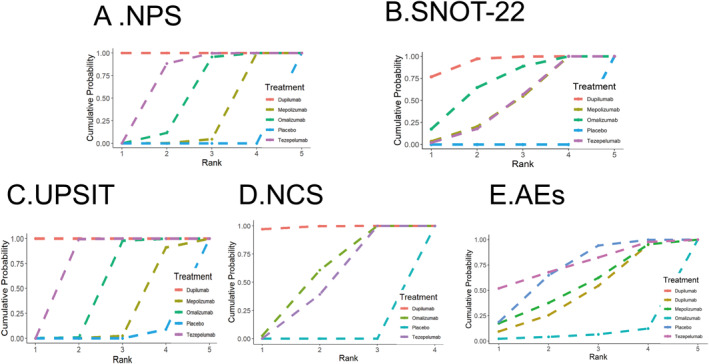
Surface under the cumulative ranking curve for the outcomes: (A) NPS (nasal polyp score); (B) SNOT‐22 (Sino‐Nasal Outcome Test‐22); (C) UPSIT (University of Pennsylvania Smell Identification Test); (D) NCS (nasal congestion score); (E) AEs (adverse events).

**FIGURE 5:**
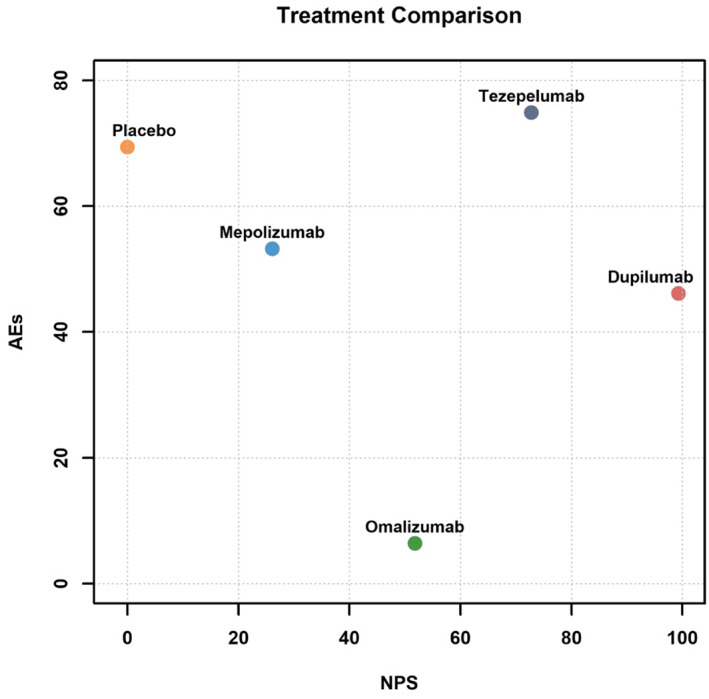
Efficacy (NPS) and safety (AEs) in all the treatments. AEs, adverse events; NPS, nasal polyp score.

The ranking of biologics according to SUCRA values is depicted in Figure 4 and detailed in Supporting Information S1: eTable 6. In this study, SUCRA‐based analysis revealed that dupilumab demonstrated superior efficacy across multiple endpoints, including NPS (NPS; SUCRA = 0.999), Quality of Life (SNOT‐22; SUCRA = 0.935), Olfactory Function (UPSIT; SUCRA = 0.999), and Nasal Congestion (NCS; SUCRA = 0.990), ranking first in all outcomes. Tezepelumab emerged as the second most effective treatment for NPS (SUCRA = 0.720) and UPSIT (SUCRA = 0.749), while ranking first in AEs (SUCRA = 0.461). Omalizumab exhibited notable efficacy in improving SNOT‐22 scores (SUCRA = 0.677) and ranked the last in AEs (SUCRA = 0.064). Mepolizumab and tezepelumab showed comparable rankings in SNOT‐22 improvement (SUCRA = 0.447 vs. 0.442, respectively) and had a moderate ranking in AEs (SUCRA = 0.532 for mepolizumab). Figure 5 illustrates the efficacy (assessed by NPS) and safety (measured by AEs) across all treatments. Dupilumab ranked first in efficacy outcomes. Tezepelumab exhibited superior efficacy compared to placebo and ranked second in terms of efficacy.

### 3.6 Sensitivity Analysis

We conducted the following post hoc sensitivity analyses to test the robustness of the findings. First, we assessed the impact of study bias risk on the results by excluding those studies categorized as having at least “some bias” (Supporting Information S1: eTable 7). Second, we removed all RCTs with a follow‐up duration of less than 24 weeks. For UPSIT, Dupilumab’s effect increased markedly (with WMD rising from 10.96 to 14.80) upon the exclusion of one study categorized as having “some bias.” In terms of ≥ 24‐week efficacy sustainability, all biologics maintained significant NPS reductions. The treatment effects of dupilumab demonstrated robustness, with both the SNOT‐22 improvement (WMD from −17.87 to −17.84) and the NCS change (WMD from −0.90 to −0.93) showing stable. Importantly, all primary efficacy endpoints and safety conclusions consistently retained their validity across sensitivity analyses, confirming the stability of the pooled results and reinforcing the reliability of clinical inferences derived from the meta‐analytic framework (Supporting Information S1: eTable 8).

## 4 Discussion

### 4.1 Principal Findings

In this study, we employed NMA to synthesize data from 13 RCTs (*n* = 2304), systematically evaluating—for the first time—the comparative efficacy of a TSLP inhibitor (tezepelumab) versus conventional Th2‐targeted biologics (dupilumab, omalizumab, and mepolizumab) in CRSwNP. Our analysis revealed that both dupilumab and tezepelumab exhibited superior efficacy across multiple endpoints. Notably, tezepelumab demonstrated exceptional performance in reducing NPS and alleviating NCS, ranking second for NPS improvement. These findings confirm the prior consensus that positioned dupilumab as the optimal therapy for CRSwNP, and provide the first evidence indicating that tezepelumab achieves comparable efficacy to omalizumab in NPS reduction and symptom relief, suggesting its potential as a novel TSLP inhibitor alternative. To ensure rigorous interpretation, we applied the latest GRADE guidelines, carefully weighing both effect magnitudes and evidence certainty.

### 4.2 Comparison With Other Studies

Dupilumab remains the most efficacious biologic for CRSwNP in this analysis, aligning with prior systematic reviews and indirect comparisons [31, 32, 33, 34]. Its superior performance likely stems from its dual inhibition of IL‐4 and IL‐13 signaling via IL‐4Rα blockade, a mechanism validated in preclinical models where IL‐4Rα knockout mice exhibited reduced mucosal eosinophilia and polyp burden in house dust mite‐induced nasal polyposis [35]. Mepolizumab also demonstrated significant efficacy in the SNOT‐22 outcome after the inclusion of new trial data (WMD: −13.47, 95% CI [−17.94, −8.99], *p* < 0.00001), contrasting with previous non‐significant findings in this measure [14]. Notably, tezepelumab addresses a critical gap in earlier NMAs, demonstrating comparable efficacy to omalizumab in NPS reduction. This phenomenon may stem from TSLP’s dual mechanism of action, which directly promotes dendritic cell‐mediated Th2 differentiation through OX40L/TSLPR signaling [36]. Preclinical evidence suggests potential indirect modulation of Th17 responses via pathways such as JAK/STAT and IL‐1β/TGF‐β, though this remains to be experimentally validated, suggesting broader anti‐inflammatory coverage beyond conventional Th2‐specific biologics [37, 38].

Clinically, tezepelumab demonstrates transformative potential, achieving a 98% reduction in polyp surgery requirements and an 88% decline in systemic corticosteroid dependence alongside sustained symptom improvement. Its safety profile includes manageable adverse events, primarily respiratory infections and nasopharyngitis, alongside notable reductions in exacerbations of chronic rhinosinusitis and comorbid asthma. However, long‐term risks—particularly recurrent respiratory infections—warrant further investigation through extended follow‐up studies and larger patient cohorts.

### 4.3 Limitations

The interpretation of findings is limited by methodological heterogeneity across studies, particularly differences in baseline population characteristics (surgical history, comorbidity burden), which may affect the validity of indirect treatment comparisons. Insufficient sample sizes for secondary endpoints further amplify these limitations. Additionally, the lack of RCTs with extended follow‐up periods (≥ 52 weeks) restricts the evaluation of long‐term outcomes, including sustained efficacy, disease recurrence, and delayed safety concerns such as cumulative infection risks. Stringent inclusion criteria in existing trials may underestimate infection risks in broader clinical populations. Notably, olfactory outcomes for tezepelumab were not examined, and observational studies were excluded to maintain methodological rigor. While SUCRA values provide useful probability estimates for intervention rankings, their clinical interpretability is constrained by two fundamental issues: the method estimates only an intervention’s relative probability of ranking highest within a specific comparison set, failing to indicate absolute superiority; more critically, it disregards both the actual magnitude of effect sizes, these factors may collectively yield misleading clinical conclusions [39, 40].

These limitations collectively constrain the interpretability of the findings and highlight the need for confirmation through rigorously designed longitudinal studies.

### 4.4 Future Research Directions

While random‐effects models and subgroup analyses may partially address heterogeneity in indirect comparisons, future head‐to‐head trials directly comparing biologic agents remain imperative. Such studies could validate efficacy differences between therapeutic classes such as TSLP inhibitors and dupilumab while clarifying their superiority in specific inflammatory endotypes. Technically, the development of multidimensional stratification models integrating noninvasive biomarkers like tissue TSLP expression levels, Th2 or non‐Th2 cytokine profiles, and clinical symptoms enhanced by machine learning algorithms is recommended to improve precision in identifying molecular endotypes of type 2 inflammation in nasal polyps. This approach could optimize biologic therapy selection. Therapeutically, investigation of TSLP inhibitors in non‐Th2 inflammatory phenotypes is warranted given their broad‐spectrum mechanisms might provide novel therapeutic pathways for mixed or neutrophilic chronic rhinosinusitis with nasal polyps. Emerging evidence supports exploring combination strategies evaluating synergies between biologics, conventional medications, surgical interventions, or upstream and downstream inhibitors, particularly in high‐risk relapse populations to define optimal therapeutic regimens.

Regarding clinical extrapolation, future research should investigate the role of biologics in pediatric nasal polyp recurrence to address efficacy gaps in younger populations while evaluating tezepelumab’s potential impact on olfactory improvement. Furthermore, the generation of long‐term real‐world evidence spanning over 5 years is critical to assess biologic durability and requires the implementation of robust safety monitoring systems.

## 5 Conclusion

This is the first study that incorporated tezepelumab into an NMA of CRSwNP biologics. Dupilumab ranked highest for efficacy (NPS, SNOT‐22, UPSIT, NCS). Tezepelumab showed comparable efficacy in NPS with omalizumab. These findings provide a critical evidence‐based foundation for advancing precision medicine strategies in CRSwNP management.

## Author Contributions

**Xi Xu:** methodology, writing – original draft, data curation. **Jinting Lin:** methodology, writing – original draft. **Minting Luo:** methodology, writing – original draft. **Qingwu Wu:** conceptualization, writing – original draft, writing – review and editing, supervision, methodology.

## Ethics Statement

This network meta‐analysis was conducted in accordance with the PRISMA guidelines. As such, ethical approval was not required since it is based exclusively on previously published studies.

## Conflicts of Interest

The authors declare no conflicts of interest.

## Supporting information


Supporting Information S1

## Data Availability

All the data are available in the manuscript and online supplement file. Further inquiries can be directed to the corresponding author.

## References

[1] R. Kratchmarov , T. Dharia , and K. Buchheit , “Clinical Efficacy and Mechanisms of Biologics for Chronic Rhinosinusitis With Nasal Polyps,” Journal of Allergy and Clinical Immunology 155, no. 5 (2025): 1401–1410, 10.1016/j.jaci.2025.03.011. 40132672 PMC12058411

[2] W. J. Fokkens , V. J. Lund , C. Hopkins , et al., “European Position Paper on Rhinosinusitis and Nasal Polyps 2020,” supplement, Rhinology 58, no. S29 (2020): 1–464, 10.4193/rhin20.600. 32077450

[3] W. W. Stevens , R. P. Schleimer , and R. C. Kern , “Chronic Rhinosinusitis With Nasal Polyps,” Journal of Allergy and Clinical Immunology: In Practice 4, no. 4 (2016): 565–572, 10.1016/j.jaip.2016.04.012. 27393770 PMC4939220

[4] L. Rudmik and T. L. Smith , “Quality of Life in Patients With Chronic Rhinosinusitis,” Current Allergy and Asthma Reports 11, no. 3 (2011): 247–252, 10.1007/s11882-010-0175-2. 21234819

[5] X. Wang , Y. Sima , Y. Zhao , et al., “Endotypes of Chronic Rhinosinusitis Based on Inflammatory and Remodeling Factors,” Journal of Allergy and Clinical Immunology 151, no. 2 (2023): 458–468, 10.1016/j.jaci.2022.10.010. 36272582

[6] P. Gevaert , T. A. Omachi , J. Corren , et al., “Efficacy and Safety of Omalizumab in Nasal Polyposis: 2 Randomized Phase 3 Trials,” Journal of Allergy and Clinical Immunology 146, no. 3 (2020): 595–605, 10.1016/j.jaci.2020.05.032. 32524991

[7] J. M. Pinto , N. Mehta , M. Ditineo , J. Wang , F. Baroody , and R. Naclerio , “A Randomized, Double‐Blind, Placebo‐Controlled Trial of Anti‐IgE for Chronic Rhinosinusitis,” Rhinology 48, no. 3 (2010): 318–324, 10.4193/rhino09.144. 21038023

[8] C. Bachert , A. R. Sousa , V. J. Lund , et al., “Reduced Need for Surgery in Severe Nasal Polyposis With Mepolizumab: Randomized Trial,” Journal of Allergy and Clinical Immunology 140, no. 4 (2017): 1024–1031.e14, 10.1016/j.jaci.2017.05.044. 28687232

[9] J. K. Han , C. Bachert , W. Fokkens , et al., “Mepolizumab for Chronic Rhinosinusitis With Nasal Polyps (SYNAPSE): A Randomised, Double‐Blind, Placebo‐Controlled, Phase 3 Trial,” Lancet Respiratory Medicine 9, no. 10 (2021): 1141–1153, 10.1016/s2213-2600(21)00097-7. 33872587

[10] P. Gevaert , N. Van Bruaene , T. Cattaert , et al., “Mepolizumab, a Humanized Anti‐IL‐5 mAb, as a Treatment Option for Severe Nasal Polyposis,” Journal of Allergy and Clinical Immunology 128, no. 5 (2011): 989–95.e1‐8, 10.1016/j.jaci.2011.07.056. 21958585

[11] C. Bachert , L. Mannent , R. M. Naclerio , et al., “Effect of Subcutaneous Dupilumab on Nasal Polyp Burden in Patients With Chronic Sinusitis and Nasal Polyposis: A Randomized Clinical Trial,” JAMA 315, no. 5 (2016): 469–479, 10.1001/jama.2015.19330. 26836729

[12] C. Bachert , J. K. Han , M. Desrosiers , et al., “Efficacy and Safety of Dupilumab in Patients With Severe Chronic Rhinosinusitis With Nasal Polyps (LIBERTY NP SINUS‐24 and LIBERTY NP SINUS‐52): Results From Two Multicentre, Randomised, Double‐Blind, Placebo‐Controlled, Parallel‐Group Phase 3 Trials,” Lancet 394, no. 10209 (2019): 1638–1650, 10.1016/s0140-6736(19)31881-1. 31543428

[13] M. A. Rank , D. K. Chu , A. Bognanni , et al., “The Joint Task Force on Practice Parameters GRADE Guidelines for the Medical Management of Chronic Rhinosinusitis With Nasal Polyposis,” Journal of Allergy and Clinical Immunology 151, no. 2 (2023): 386–398, 10.1016/j.jaci.2022.10.026. 36370881

[14] Q. Wu , Y. Zhang , W. Kong , et al., “Which Is the Best Biologic for Nasal Polyps: Dupilumab, Omalizumab, or Mepolizumab? A Network Meta‐Analysis,” International Archives of Allergy and Immunology 183, no. 3 (2022): 279–288, 10.1159/000519228. 34607329

[15] Q. Wang , Q. Sun , Q. Chen , H. Li , D. Liu , and Q. Wu , “Efficacy and Safety of Anti‐Interleukin‐5 Therapies in Chronic Rhinosinusitis With Nasal Polyps: A Systematic Review and Meta‐Analysis of Randomized Controlled Trials,” International Archives of Allergy and Immunology 183, no. 7 (2022): 732–743, 10.1159/000521859. 35108711

[16] Q. Wu , L. Yuan , H. Qiu , et al., “Efficacy and Safety of Omalizumab in Chronic Rhinosinusitis With Nasal Polyps: A Systematic Review and Meta‐Analysis of Randomised Controlled Trials,” BMJ Open 11, no. 9 (2021): e047344, 10.1136/bmjopen-2020-047344. PMC8420736 34479933

[17] G. M. Gauvreau , P. M. O’Byrne , L. P. Boulet , et al., “Effects of an Anti‐TSLP Antibody on Allergen‐Induced Asthmatic Responses,” New England Journal of Medicine 370, no. 22 (2014): 2102–2110, 10.1056/nejmoa1402895. 24846652

[18] G. Marone , G. Spadaro , M. Braile , et al., “Tezepelumab: A Novel Biological Therapy for the Treatment of Severe Uncontrolled Asthma,” Expert Opinion on Investigational Drugs 28, no. 11 (2019): 931–940, 10.1080/13543784.2019.1672657. 31549891

[19] V. Soumelis , P. A. Reche , H. Kanzler , et al., “Human Epithelial Cells Trigger Dendritic Cell Mediated Allergic Inflammation by Producing TSLP,” Nature Immunology 3, no. 7 (2002): 673–680, 10.1038/ni805. 12055625

[20] B. Liao , P. P. Cao , M. Zeng , et al., “Interaction of Thymic Stromal Lymphopoietin, IL‐33, and Their Receptors in Epithelial Cells in Eosinophilic Chronic Rhinosinusitis With Nasal Polyps,” Allergy 70, no. 9 (2015): 1169–1180, 10.1111/all.12667. 26095319

[21] K. Verstraete , F. Peelman , H. Braun , et al., “Structure and Antagonism of the Receptor Complex Mediated by Human TSLP in Allergy and Asthma,” Nature Communications 8, no. 1 (2017): 14937, 10.1038/ncomms14937. PMC5382266 28368013

[22] G. M. Gauvreau , R. Sehmi , C. S. Ambrose , and J. M. Griffiths , “Thymic Stromal Lymphopoietin: Its Role and Potential as a Therapeutic Target in Asthma,” Expert Opinion on Therapeutic Targets 24, no. 8 (2020): 777–792, 10.1080/14728222.2020.1783242. 32567399

[23] B. J. Lipworth , J. K. Han , M. Desrosiers , et al., “Tezepelumab in Adults With Severe Chronic Rhinosinusitis With Nasal Polyps,” New England Journal of Medicine 392, no. 12 (2025): 1178–1188, 10.1056/nejmoa2414482. 40106374

[24] A. Izcovich , D. K. Chu , R. A. Mustafa , G. Guyatt , and R. Brignardello‐Petersen , “A Guide and Pragmatic Considerations for Applying GRADE to Network meta‐analysis,” BMJ 381 (2023): e074495, 10.1136/bmj-2022-074495. 37369385

[25] B. Hutton , G. Salanti , D. M. Caldwell , et al., “The PRISMA Extension Statement for Reporting of Systematic Reviews Incorporating Network Meta‐Analyses of Health Care Interventions: Checklist and Explanations,” Annals of Internal Medicine 162, no. 11 (2015): 777–784, 10.7326/m14-2385. 26030634

[26] W. W. Busse , J. F. Maspero , K. F. Rabe , et al., “Liberty Asthma QUEST: Phase 3 Randomized, Double‐Blind, Placebo‐Controlled, Parallel‐Group Study to Evaluate Dupilumab Efficacy/Safety in Patients With Uncontrolled, Moderate‐to‐Severe Asthma,” Advances in Therapy 35, no. 5 (2018): 737–748, 10.1007/s12325-018-0702-4. 29725983 PMC5960488

[27] K. F. Rabe , P. Nair , G. Brusselle , et al., “Efficacy and Safety of Dupilumab in Glucocorticoid‐Dependent Severe Asthma,” New England Journal of Medicine 378, no. 26 (2018): 2475–2485, 10.1056/nejmoa1804093. 29782224

[28] P. Gevaert , L. Calus , T. Van Zele , et al., “Omalizumab Is Effective in Allergic and Nonallergic Patients With Nasal Polyps and Asthma,” Journal of Allergy and Clinical Immunology 131, no. 1 (2013): 110–116.e1, 10.1016/j.jaci.2012.07.047. 23021878

[29] G. Salanti , C. Del Giovane , A. Chaimani , D. M. Caldwell , and J. P. T. Higgins , “Evaluating the Quality of Evidence From a Network Meta‐Analysis,” PLoS One 9, no. 7 (2014): e99682, 10.1371/journal.pone.0099682. 24992266 PMC4084629

[30] G. L. Chupp , E. S. Bradford , F. C. Albers , et al., “Efficacy of Mepolizumab Add‐on Therapy on Health‐Related Quality of Life and Markers of Asthma Control in Severe Eosinophilic Asthma (MUSCA): A Randomised, Double‐Blind, Placebo‐Controlled, Parallel‐Group, Multicentre, Phase 3b Trial,” Lancet Respiratory Medicine 5, no. 5 (2017): 390–400, 10.1016/s2213-2600(17)30125-x. 28395936

[31] L. Y. Chong , P. Piromchai , S. Sharp , et al., “Biologics for Chronic Rhinosinusitis,” Cochrane Database of Systematic Reviews 3, no. 3 (2021): Cd013513, 10.1002/14651858.CD013513.pub3. 33710614 PMC8094915

[32] S. Cai , S. Xu , H. Lou , and L. Zhang , “Comparison of Different Biologics for Treating Chronic Rhinosinusitis With Nasal Polyps: A Network Analysis,” Journal of Allergy and Clinical Immunology: In Practice 10, no. 7 (2022): 1876–1886.e7, 10.1016/j.jaip.2022.02.034. 35272073

[33] P. Oykhman , F. A. Paramo , J. Bousquet , D. W. Kennedy , R. Brignardello‐Petersen , and D. K. Chu , “Comparative Efficacy and Safety of Monoclonal Antibodies and Aspirin Desensitization for Chronic Rhinosinusitis With Nasal Polyposis: A Systematic Review and Network Meta‐Analysis,” Journal of Allergy and Clinical Immunology 149, no. 4 (2022): 1286–1295, 10.1016/j.jaci.2021.09.009. 34543652

[34] A. T. Peters , J. K. Han , P. Hellings , et al., “Indirect Treatment Comparison of Biologics in Chronic Rhinosinusitis With Nasal Polyps,” Journal of Allergy and Clinical Immunology: In Practice 9, no. 6 (2021): 2461–2471.e5, 10.1016/j.jaip.2021.01.031. 33548517

[35] A. Le Floc'H , J. Allinne , K. Nagashima , et al., “Dual Blockade of IL‐4 and IL‐13 With Dupilumab, an IL‐4Rα Antibody, Is Required to Broadly Inhibit Type 2 Inflammation,” Allergy 75, no. 5 (2020): 1188–1204, 10.1111/all.14151. 31838750 PMC7317958

[36] Y. Liang , B. Yu , J. Chen , et al., “Thymic Stromal Lymphopoietin Epigenetically Upregulates Fc Receptor γ Subunit‐Related Receptors on Antigen‐Presenting Cells and Induces T(H)2/T(H)17 Polarization Through Dectin‐2,” Journal of Allergy and Clinical Immunology 144, no. 4 (2019): 1025–1035.e7, 10.1016/j.jaci.2019.06.011. 31251950

[37] F. Han , H. Guo , L. Wang , et al., “TSLP Produced by Aspergillus Fumigatus‐Stimulated DCs Promotes a Th17 Response Through the JAK/STAT Signaling Pathway in Fungal Keratitis,” Investigative Ophthalmology & Visual Science 61, no. 14 (2020): 24, 10.1167/iovs.61.14.24. PMC7757613 33346778

[38] S. I. Bogiatzi , M. Guillot‐Delost , A. Cappuccio , et al., “Multiple‐Checkpoint Inhibition of Thymic Stromal Lymphopoietin‐Induced TH2 Response by TH17‐Related Cytokines,” Journal of Allergy and Clinical Immunology 130, no. 1 (2012): 233–240.e5, 10.1016/j.jaci.2012.04.038. 22664159

[39] G. Salanti , A. E. Ades , and J. P. Ioannidis , “Graphical Methods and Numerical Summaries for Presenting Results From Multiple‐Treatment Meta‐Analysis: An Overview and Tutorial,” Journal of Clinical Epidemiology 64, no. 2 (2011): 163–171, 10.1016/j.jclinepi.2010.03.016. 20688472

[40] L. Mbuagbaw , B. Rochwerg , R. Jaeschke , et al., “Approaches to Interpreting and Choosing the Best Treatments in Network Meta‐Analyses,” Systematic Reviews 6, no. 1 (2017): 79, 10.1186/s13643-017-0473-z. 28403893 PMC5389085

