# Supplementary material for: Which Is the Best Biologic for Nasal Polyps: An Updated Network Meta‐Analysis
Source: Clin Transl Allergy. 2025 Nov 3;15(11):e70114. doi: 10.1002/clt2.70114 (PMC12580786; doi:10.1002/clt2.70114)
Supplement: Supplementary file 1 — Supporting Information S1 [file CLT2-15-e70114-s001.docx]

**eTable 1**. Search strategies

| **PubMed** |
| --- |
| 1.nasal polyps[MeSH Terms]  2.((nasal polyp*[Title/Abstract]) OR (nose polyp*[Title/Abstract])) OR (nasal papilloma[Title/Abstract])  3. (#1) OR (#2)  4.sinusitis[MeSH Terms]  5.((((((CRSwNP[Title/Abstract]) OR (chronic rhinosinusitis[Title/Abstract])) OR (rhinopolyp*[Title/Abstract])) OR (sinus Infection*[Title/Abstract])) OR (Rhinitis[Title/Abstract])) OR (sphenoid* sinusitis[Title/Abstract])) OR (pansinusitis[Title/Abstract])  6.(#4) OR (#5)  7.(#3) OR (#6)  8.dupilumab[Supplementary Concept]  9. (((dupilumab[Title/Abstract]) OR (Dupixent[Title/Abstract])) OR (Anti-IL-4Rα mAb[Title/Abstract])) OR (Anti-IL-4Rα monoclonal antibody[Title/Abstract])  10. (#8) OR (#9)  11. mepolizumab[Supplementary Concept]  12.((((mepolizumab[Title/Abstract]) OR (Bosatria[Title/Abstract])) OR (Nucala[Title/Abstract])) OR (Anti-IL-5 mAb[Title/Abstract])) OR (Anti-IL-5 monoclonal antibody[Title/Abstract])  13. (#11) OR (#12)  14. omalizumab[MeSH Terms]  15.(((Xolair[Title/Abstract]) OR (anti-IgE monoclonal antibody[Title/Abstract])) OR (anti-IgE mAb[Title/Abstract])) OR (anti-IgE antibody[Title/Abstract])  16.(#14) OR (#15)  17. tezepelumab[Supplementary Concept]  18.(((((tezepelumab[Title/Abstract]) OR (tezspire[Title/Abstract])) OR (tezepelumab-ekko[Title/Abstract])) OR (Anti-TSLP antibody[Title/Abstract])) OR ( Anti-TSLP mAb[Title/Abstract])) OR (Anti-TSLP monoclonal antibody[Title/Abstract])  19. (#17) OR (#18)  20.(((((lebrikizumab[Title/Abstract]) OR (tralokinumab[Title/Abstract])) OR (anti-IL-13 monoclonal antibodies[Title/Abstract])) OR (anti-IL-13 mAb[Title/Abstract])) OR (reslizumab[Title/Abstract])) OR (benralizumab[Title/Abstract])  21.antibodies, monoclonal[MeSH Terms]  22. (#20) OR (#21)  23.#10 OR #13 OR #16 OR #19 OR #22  24.#7 AND #23 |
| **Cochrane Library** |
| 1. MeSH descriptor: [Nasal Polyps] explode all trees 609  2. (nasal polyp*):ti,ab,kw OR (nose polyp*):ti,ab,kw OR (nasi papilloma):ti,ab,kw OR (nasal papilloma):ti,ab,kw OR (nasi polyposis):ti,ab,kw 1735  3.#1 OR #2 1735  4. MeSH descriptor: [Sinusitis] explode all trees 1484  5.(CRSwNP):ti,ab,kw OR (chronic rhinosinusitis):ti,ab,kw OR (rhinopolyp*):ti,ab,kw OR (sinus Infection*):ti,ab,kw OR (rhinitis):ti,ab,kw 13331  6. #4 OR #5 13863  7. #3 OR #6 14503  8. (dupilumab):ti,ab,kw OR (Dupixent):ti,ab,kw OR (Anti-IL-4Rα mAb):ti,ab,kw OR (Anti-IL-4Rα monoclonal antibody):ti,ab,kw 1346  9.(mepolizumab):ti,ab,kw OR (Bosatria):ti,ab,kw OR (Nucala):ti,ab,kw OR (Anti-IL-5 mAb):ti,ab,kw OR (Anti-IL-5 monoclonal antibody):ti,ab,kw 503  10. MeSH descriptor: [Omalizumab] explode all trees 402  11.(Xolair):ti,ab,kw OR (anti-IgE monoclonal antibody):ti,ab,kw OR (anti-IgE mAb):ti,ab,kw OR (anti-IgE antibody):ti,ab,kw 209  12. MeSH descriptor: [Antibodies, Monoclonal] explode all trees 21960  13.(lebrikizumab):ti,ab,kw OR (tralokinumab):ti,ab,kw OR (anti-IL-13 mAb):ti,ab,kw OR (reslizumab):ti,ab,kw OR (benralizumab):ti,ab,kw 805  14.(tezepelumab):ti,ab,kw OR (tezspire):ti,ab,kw OR (Anti-TSLP antibody):ti,ab,kw OR (Anti-TSLP mAb):ti,ab,kw 252  15.#8 OR #9 OR #10 OR #11 OR #12 OR #13 OR #14 24314  16.#7 AND #15 550 |
| **Embase** |
| 1. 'nose polyp'/exp  2.'nasal polyp*':ab,ti OR 'nose polyp*':ab,ti OR 'nasi papilloma':ab,ti OR 'nasal papilloma':ab,ti OR 'nasi polyposis':ab,ti  3. #1 OR #2  4. 'sinusitis'/exp  5.crswnp:ab,ti OR 'chronic rhinosinusitis':ab,ti OR rhinopolyp*:ab,ti OR 'sinus infection*':ab,ti OR rhinitis:ab,ti OR 'sphenoid* sinusitis':ab,ti  6. #4 OR #5  7.'dupilumab'/exp  8.dupilumab:ab,ti OR dupixent:ab,ti OR 'anti-il-4r mab':ab,ti OR 'anti-il-4r monoclonal antibody':ab,ti  9. #7 OR #8  10.'mepolizumab'/exp  11.mepolizumab:ab,ti OR bosatria:ab,ti OR nucala:ab,ti OR 'anti-il-5 mab':ab,ti OR 'anti-il-5 monoclonal antibody':ab,ti  12.#10 OR #11  13.'omalizumab'/exp  14.xolair:ab,ti OR 'anti-ige monoclonal antibody':ab,ti OR 'anti-ige mab':ab,ti OR 'anti-ige antibody':ab,ti  15.#13 OR #14  16. 'monoclonal antibody'/exp  17.lebrikizumab:ab,ti OR tralokinumab:ab,ti OR 'anti-il-13 mab':ab,ti OR reslizumab:ab,ti OR 'benralizumab'/exp OR benralizumab  18. #16 OR #17  19. 'tezepelumab'/exp  20.tezepelumab:ab,ti OR tezspire:ab,ti OR 'tezepelumab-ekko':ab,ti OR 'anti -tslp antibody':ab,ti OR 'anti -tslp mab':ab,ti OR 'anti -tslp monoclonal antibody':ab,ti  21. #19 OR #20  22. #3 OR #6  23.#9 OR #12 OR #15 OR #18 OR #21  24.#22 AND #23  25.#24 AND [medline]/lim  26.#24 NOT #25 |
| **Web of Science** |
| 1. ((((TS=(nasal polyp*)) OR TS=(nose polyp*)) OR TS=(nasi papilloma)) OR TS=(nasal papilloma)) OR TS=(nasi polyposis) and Preprint Citation Index (Exclude – Database)  2. ((((((TS=(sinusitis)) OR TS=(CRSwNP)) OR TS=(chronic rhinosinusitis)) OR TS=(rhinopolyp*)) OR TS=(sinus Infection*)) OR TS=(rhinitis)) OR TS=(sphenoid* sinusitis) and Preprint Citation Index (Exclude – Database)  3. #2 OR #1 and Preprint Citation Index (Exclude – Database)  4. (((TS=(dupilumab)) OR TS=(Dupixent)) OR TS=(Anti-IL-4Rα mAb)) OR TS=(Anti-IL-4Rα monoclonal antibody) and Preprint Citation Index (Exclude – Database)  5.((((TS=(mepolizumab)) OR TS=(Bosatria)) OR TS=(Nucala)) OR TS=(Anti-IL-5 mAb)) OR TS=(Anti-IL-5 monoclonal antibody) and Preprint Citation Index (Exclude – Database)  6.((((TS=(omalizumab)) OR TS=(Xolair)) OR TS=(anti-IgE monoclonal antibody)) OR TS=(anti-IgE mAb)) OR TS=(anti-IgE antibody) and Preprint Citation Index (Exclude – Database)  7. (((((TS=(monoclonal antibody)) OR TS=(ebrikizumab)) OR TS=(tralokinumab)) OR TS=(anti-IL-13 mAb)) OR TS=(reslizumab)) OR TS=(benralizumab) and Preprint Citation Index (Exclude – Database)  8.(((((TS=(tezepelumab)) OR TS=(tezspire)) OR TS=(tezepelumab-ekko)) OR TS=(anti tslp antibody)) OR TS=(anti tslp mab)) OR TS=(anti tslp monoclonal antibody) and Preprint Citation Index (Exclude – Database)  9. #4 OR #5 OR #6 OR #7 OR #8 and Preprint Citation Index (Exclude – Database)  10. #9 AND #3 and Preprint Citation Index (Exclude – Database) |

**eTable 2**. Characteristics of outcomes in network meta-analysis

| **Outcomes** | **Size of study**  **population** | **Including**  **RCTs, n** | **Scale Range** | **Directionality** | **MCID** |  |
| --- | --- | --- | --- | --- | --- | --- |
| NPS | 2005 | 11 | 0-8 | Decreas (reduced polyp mass) | ≥1-point reduction ^[6, 8-11, 23]^  ≥2-point reduction ^[6, 9, 11, 12, 23]^ |  |
| SNOT-22 | 2203 | 10 | 0-110 | Decrease (improved symptoms/quality of life) | ≥8.9-point reduction ^[6, 23]^ |  |
| *UPSIT | 1864 | 7 | 0-40 | Increase (improved olfactory function) | - |  |
| NCS | 1480 | 7 | 0-3 | Decrease (relieved congestion) | ≥1-point reduction ^[9]^ |  |
| AEs | 2034 | 11 | Dichotomous | Decrease (lower risk) | - |  |

MCID, minimal clinically important difference; NPS, nasal polyp score; SNOT-22, Sino-Nasal Outcome Test-22; UPSIT, University of Pennsylvania Smell Identification Test; NCS, nasal congestion score; AEs, adverse events; *UPSIT interpretation lacked predefined clinical thresholds.

Gevaert2020 ^[6]^: Omalizumab compared to placebo: 56.3% vs 28.7% for >1-point NPS reduction. 31.3% vs 11.6% for >2-point NPS reduction, and 44.4% vs 21.4% for the >1-point NCS reduction threshold.

Bachert2017^[8]^: Mepolizumab compared to placebo: 50.0% vs 27.0% for >1-point NPS reduction.

Han2021(SYNAPSE) ^[9]^: Mepolizumab compared to placebo: 50.0% vs 28.0% for >1-point NPS reduction. 36.0% vs 13.0% for >2-point NPS reduction, and 73.0% vs 54.0% for the≥8.9-point reduction in SNOT-22.

Gevaert2011^[10]^: Mepolizumab compared to placebo: 60.0% vs 10.0% for >1-point NPS reduction.

Bachert2016^[11]^: Dupilumab compared to placebo: 70.0% vs 20.0% for >1-point NPS reduction. 53.0% vs 10.0% for >2-point NPS reduction.

Bachert2019 ^[12]^: Dupilumab compared to placebo: at 24 weeks, in the SINUS-24, 46.0% vs 5.0% for >2-point NPS reduction; in the SINUS-52 trial, 46.0% vs 1.0% for >2-point NPS reduction.

Lipworth2025^[23]^: Tezepelumab compared to placebo: 79.3% vs 31.7% for >1-point NPS reduction. 63.5% vs 19.0% for >2-point NPS reduction, and 73.4% vs 33.2% for the >1-point NCS reduction threshold.

**eTable 3**. Summary of certainty of the evidence (GRADE)

**eTable 3-A** Summary of findings for NPS: GRADE of direct and indirect comparisons of monoclonal antibodies

| **Certainty assessment** | | | | | | | **№ of patients** | | **Effect** | | **Certainty** | **Importance** |
| --- | --- | --- | --- | --- | --- | --- | --- | --- | --- | --- | --- | --- |
| **№ of studies** | **Study design** | **Risk of bias** | **Inconsistency** | **Indirectness** | **Imprecision** | **Other considerations** | **Biologics** | **Placebo** | **Relative (95% CI)** | **Absolute (95% CI)** |  |  |
| **Omalizumab vs Placebo** | | | | | | | | | | | | |
| 4 | randomised trials | not serious | serious^a^ | not serious | not serious | none | 156 | 146 | - | WMD **0 -1.25**  (1.52 lower to 0.97 lower) | ⨁⨁⨁◯ Moderate^a^ | IMPORTANT |
| **Dupilumab vs Placebo** | | | | | | | | | | | | |
| 3 | randomised trials | not serious | not serious | not serious | not serious | none | 468 | 316 | - | WMD **2.16 lower** (2.44 lower to 1.89 lower) | ⨁⨁⨁⨁ High | IMPORTANT |
| **Mepolizumab vs Placebo** | | | | | | | | | | | | |
| 3 | randomised trials | not serious | not serious | not serious | not serious | none | 268 | 243 | - | WMD **0.9 lower** (1.19 lower to 0.62 lower) | ⨁⨁⨁⨁ High | IMPORTANT |
| **Tezepelumab vs Placebo** | | | | | | | | | | | | |
| 1 | randomised trials | not serious | not serious | not serious | not serious | none | 203 | 205 | - | WMD **1.5 lower** (1.81 lower to 1.19 lower) | ⨁⨁⨁⨁ High | IMPORTANT |
| **Dupilumab vs Mepolizumab** | | | | | | | | | | | | |
| 0 | randomised trials | not serious | not serious | not serious | not serious | none | - | - | - | WMD **1.26 lower** (1.65 lower to 0.86 lower) | ⨁⨁⨁⨁ High | IMPORTANT |
| **Dupilumab vs Omalizumab** | | | | | | | | | | | | |
| 0 | randomised trials | not serious | serious^b^ | not serious | not serious | none | - | - | - | WMD **0.92 lower** (1.31 lower to 0.53 lower) | ⨁⨁⨁◯ Moderate^b^ | IMPORTANT |
| **Dupilumab vs Tezepelumab** | | | | | | | | | | | | |
| 0 | randomised trials | not serious | not serious | not serious | not serious | none | - | - | - | WMD **0.67 lower** (1.08 lower to 0.25 lower) | ⨁⨁⨁⨁ High | IMPORTANT |
| **Mepolizumab vs Omalizumab** | | | | | | | | | | | | |
| 0 | randomised trials | not serious | serious^b^ | not serious | serious^c^ | none | - | - | - | WMD **0.34 higher** (0.05 lower to 0.73 higher) | ⨁⨁◯◯ Low^b,c^ | IMPORTANT |
| **Mepolizumab vs Tezepelumab** | | | | | | | | | | | | |
| 0 | randomised trials | not serious | not serious | not serious | not serious | none | - | - | - | WMD **0.59 higher** (0.17 higher to 1.01 higher) | ⨁⨁⨁⨁ High | IMPORTANT |
| **Omalizumab vs Tezepelumab** | | | | | | | | | | | | |
| 0 | randomised trials | not serious | serious^b^ | not serious | serious^c^ | none | - | - | - | WMD **0.25 higher** (0.16 lower to 0.67 higher) | ⨁⨁◯◯ Low^b,c^ | IMPORTANT |

**CI:** confidence interval; **WMD:** weighted mean difference

**Explanations**

a. Direct evidence downgraded -1 due to heterogeneity.

b. Indirect evidence downgraded -1 due to heterogeneity.

c. Indirect evidence downgraded -1 due to imprecision.

**Grade Definition**

**High:** Further research is very unlikely to change our confidence in the estimate of effect.

**Moderate:** Further research is likely to have an important impact on our confidence in the estimate of effect and may change the estimate.

**Low:** Further research is very likely to have an important impact on our confidence in the estimate of effect and is likely to change the estimate.

**Very low**: Any estimate of effect is very uncertain

**eTable 3-B** Summary of findings for SNOT-22: GRADE of direct and indirect comparisons of monoclonal antibodies

| **Certainty assessment** | | | | | | | **№ of patients** | | **Effect** | | **Certainty** | **Importance** |
| --- | --- | --- | --- | --- | --- | --- | --- | --- | --- | --- | --- | --- |
| **№ of studies** | **Study design** | **Risk of bias** | **Inconsistency** | **Indirectness** | **Imprecision** | **Other considerations** | **Biologics** | **placebo** | **Relative (95% CI)** | **Absolute (95% CI)** |  |  |
| **Omalizumab vs Placebo** | | | | | | | | | | | | |
| 2 | randomised trials | not serious | not serious | not serious | not serious | none | 134 | 131 | - | WMD **15.62 lower** (19.79 lower to 11.45 lower) | ⨁⨁⨁⨁ High | IMPORTANT |
| **Dupilumab vs Placebo** | | | | | | | | | | | | |
| 5 | randomised trials | not serious | serious^a^ | serious^b^ | not serious | none | 622 | 427 | - | WMD **17.87 lower** (20.34 lower to 15.39 lower) | ⨁⨁◯◯ Low^a,b^ | IMPORTANT |
| **Mepolizumab vs Placebo** | | | | | | | | | | | | |
| 2 | randomised trials | not serious | not serious | not serious | not serious | none | 248 | 233 | - | WMD **13.47 lower** (17.94 lower to 8.99 lower) | ⨁⨁⨁⨁ High | IMPORTANT |
| **Tezepelumab vs Placebo** | | | | | | | | | | | | |
| 1 | randomised trials | not serious | not serious | not serious | not serious | none | 203 | 205 | - | WMD **13.5 lower** (17.31 lower to 9.69 lower) | ⨁⨁⨁⨁ High | IMPORTANT |
| **Dupilumab vs Mepolizumab** | | | | | | | | | | | | |
| 0 | randomised trials | not serious | serious^c^ | serious^d^ | serious^e^ | none | - | - | - | WMD **4.4 lower** (9.54 lower to 0.76 higher) | ⨁◯◯◯ Very low^c,d,e^ | IMPORTANT |
| **Dupilumab vs Omalizumab** | | | | | | | | | | | | |
| 0 | randomised trials | not serious | serious^c^ | serious^d^ | serious^e^ | none | - | - | - | WMD **2.24 lower** (7.09 lower to 2.64 higher) | ⨁◯◯◯ Very low^c,d,e^ | IMPORTANT |
| **Dupilumab vs Tezepelumab** | | | | | | | | | | | | |
| 0 | randomised trials | not serious | serious^c^ | serious^d^ | serious^e^ | none | - | - | - | WMD **4.35 lower** (8.89 lower to 0.17 higher) | ⨁◯◯◯ Very low^c,d,e^ | IMPORTANT |
| **Mepolizumab vs Omalizumab** | | | | | | | | | | | | |
| 0 | randomised trials | not serious | not serious | not serious | serious^e^ | none | - | - | - | WMD **2.18 higher** (3.97 lower to 8.29 higher) | ⨁⨁⨁◯ Moderate^e^ | IMPORTANT |
| **Mepolizumab vs Tezepelumab** | | | | | | | | | | | | |
| 0 | randomised trials | not serious | not serious | not serious | serious^e^ | none | - | - | - | WMD **0.033 higher** (5.87 lower to 5.96 higher) | ⨁⨁⨁◯ Moderate^e^ | IMPORTANT |
| **Omalizumab vs Tezepelumab** | | | | | | | | | | | | |
| 0 | randomised trials | not serious | not serious | not serious | serious^e^ | none | - | - | - | WMD **2.12 lower** (7.77 lower to 3.53 higher) | ⨁⨁⨁◯ Moderate^e^ | IMPORTANT |

**CI:** confidence interval; **WMD:** weighted mean difference

**Explanations**

a. Direct evidence downgraded -1 due to heterogeneity.

b. Direct evidence downgraded -1 due to population heterogeneity

c. Indirect evidence downgraded -1 due to heterogeneity.

d. Indirect evidence downgraded -1 due to population heterogeneity

e. Indirect evidence downgraded -1 due to imprecision.

**Grade Definition**

**High:** Further research is very unlikely to change our confidence in the estimate of effect.

**Moderate:** Further research is likely to have an important impact on our confidence in the estimate of effect and may change the estimate.

**Low:** Further research is very likely to have an important impact on our confidence in the estimate of effect and is likely to change the estimate.

**Very low**: Any estimate of effect is very uncertain

**eTable 3**-C Summary of findings for UPSIT: GRADE of direct and indirect comparisons of monoclonal antibodies

| **Certainty assessment** | | | | | | | **№ of patients** | | **Effect** | | **Certainty** | **Importance** |
| --- | --- | --- | --- | --- | --- | --- | --- | --- | --- | --- | --- | --- |
| **№ of studies** | **Study design** | **Risk of bias** | **Inconsistency** | **Indirectness** | **Imprecision** | **Other considerations** | **Biologics** | **placebo** | **Relative (95% CI)** | **Absolute (95% CI)** |  |  |
| **Omalizumab vs Placebo** | | | | | | | | | | | | |
| 2 | randomised trials | not serious | not serious | not serious | not serious | none | 134 | 131 | - | WMD **3.84 higher** (2.19 higher to 5.5 higher) | ⨁⨁⨁⨁ High | IMPORTANT |
| **Dupilumab vs Placebo** | | | | | | | | | | | | |
| 3 | randomised trials | not serious | serious^a^ | not serious | not serious | none | 468 | 316 | - | WMD **10.95 higher** (9.73 higher to 12.16 higher) | ⨁⨁⨁◯ Moderate^a^ | IMPORTANT |
| **Mepolizumab vs Placebo** | | | | | | | | | | | | |
| 1 | randomised trials | not serious | not serious | not serious | serious^b^ | none | 206 | 201 | - | WMD **1.3 higher** (0.59 lower to 3.19 higher) | ⨁⨁⨁◯ Moderate^b^ | IMPORTANT |
| **Tezepelumab vs Placebo** | | | | | | | | | | | | |
| 1 | randomised trials | not serious | not serious | not serious | not serious | none | 203 | 205 | - | WMD **6.7 higher** (5.21 higher to 8.19 higher) | ⨁⨁⨁⨁ High | IMPORTANT |
| **Dupilumab vs Mepolizumab** | | | | | | | | | | | | |
| 0 | randomised trials | not serious | serious^c^ | not serious | serious^d^ | none | - | - | - | WMD **9.19 higher** (6.93 higher to 11.45 higher) | ⨁⨁◯◯ Low^c,d^ | IMPORTANT |
| **Dupilumab vs Omalizumab** | | | | | | | | | | | | |
| 0 | randomised trials | not serious | serious^c^ | not serious | not serious | none | - | - | - | WMD **6.63 higher** (4.57 higher to 8.69 higher) | ⨁⨁⨁◯ Moderate^c^ | IMPORTANT |
| **Dupilumab vs Tezepelumab** | | | | | | | | | | | | |
| 0 | randomised trials | not serious | serious^c^ | not serious | not serious | none | - | - | - | WMD **3.78 higher** (1.85 higher to 5.71 higher) | ⨁⨁⨁◯ Moderate^c^ | IMPORTANT |
| **Mepolizumab vs Omalizumab** | | | | | | | | | | | | |
| 0 | randomised trials | not serious | not serious | not serious | serious^d^ | none | - | - | - | WMD **2.56 lower** (5.06 lower to 0.05 lower) | ⨁⨁⨁◯ Moderate^d^ | IMPORTANT |
| **Mepolizumab vs Tezepelumab** | | | | | | | | | | | | |
| 0 | randomised trials | not serious | not serious | not serious | serious^d^ | none | - | - | - | WMD **5.42 lower** (7.83 lower to 2.99 lower) | ⨁⨁⨁◯ Moderate^d^ | IMPORTANT |
| **Omalizumab vs Tezepelumab** | | | | | | | | | | | | |
| 0 | randomised trials | not serious | not serious | not serious | not serious | none | - | - | - | WMD **2.85 lower** (5.08 lower to 0.63 lower) | ⨁⨁⨁⨁ High | IMPORTANT |

**CI:** confidence interval; **WMD:** weighted mean difference e

**Explanations**

a. Direct evidence downgraded -1 due to heterogeneity.

b. Direct evidence downgraded -1 due to imprecision.

c. Indirect evidence downgraded -1 due to heterogeneity.

d. Indirect evidence downgraded -1 due to imprecision.

**Grade Definition**

**High:** Further research is very unlikely to change our confidence in the estimate of effect.

**Moderate:** Further research is likely to have an important impact on our confidence in the estimate of effect and may change the estimate.

**Low:** Further research is very likely to have an important impact on our confidence in the estimate of effect and is likely to change the estimate.

**Very low**: Any estimate of effect is very uncertain

**eTable 3**-D Summary of findings for NCS: GRADE of direct and indirect comparisons of monoclonal antibodies

| **Certainty assessment** | | | | | | | **№ of patients** | | **Effect** | | **Certainty** | **Importance** |
| --- | --- | --- | --- | --- | --- | --- | --- | --- | --- | --- | --- | --- |
| **№ of studies** | **Study design** | **Risk of bias** | **Inconsistency** | **Indirectness** | **Imprecision** | **Other considerations** | **Biologics** | **placebo** | **Relative (95% CI)** | **Absolute (95% CI)** |  |  |
| **Omalizumab vs Placebo** | | | | | | | | | | | | |
| 3 | randomised trials | not serious | serious^a^ | not serious | not serious | none | 149 | 139 | - | WMD **0.67 lower** (0.87 lower to 0.48 lower) | ⨁⨁⨁◯ Moderate^a^ | IMPORTANT |
| **Dupilumab vs Placebo** | | | | | | | | | | | | |
| 3 | randomised trials | not serious | not serious | not serious | not serious | none | 468 | 316 | - | WMD **0.9 lower** (1.04 lower to 0.77 lower) | ⨁⨁⨁⨁ High | IMPORTANT |
| **Tezepelumab vs Placebo** | | | | | | | | | | | | |
| 1 | randomised trials | not serious | not serious | not serious | not serious | none | 203 | 205 | - | WMD **0.64 lower** (0.78 lower to 0.5 lower) | ⨁⨁⨁⨁ High | IMPORTANT |
| **Dupilumab vs Omalizumab** | | | | | | | | | | | | |
| 0 | randomised trials | not serious | serious^b^ | not serious | serious^c^ | none | - | - | - | WMD **0.23 lower** (0.46 lower to 0.002 higher) | ⨁⨁◯◯ Low^b,c^ | IMPORTANT |
| **Dupilumab vs Tezepelumab** | | | | | | | | | | | | |
| 0 | randomised trials | not serious | not serious | not serious | not serious | none | - | - | - | WMD **0.26 lower** (0.45 lower to 0.069 lower) | ⨁⨁⨁⨁ High | IMPORTANT |
| **Omalizumab vs Tezepelumab** | | | | | | | | | | | | |
| 0 | randomised trials | not serious | serious^b^ | not serious | serious^c^ | none | - | - | - | WMD **0.03 lower** (0.27 lower to 0.2 higher) | ⨁⨁◯◯ Low^b,c^ | IMPORTANT |

**CI:** confidence interval; **WMD:** weighted mean difference

**Explanations**

a. Direct evidence downgraded -1 due to heterogeneity.

b. Indirect evidence downgraded -1 due to heterogeneity.

c. Indirect evidence downgraded -1 due to imprecision.

**Grade Definition**

**High:** Further research is very unlikely to change our confidence in the estimate of effect.

**Moderate:** Further research is likely to have an important impact on our confidence in the estimate of effect and may change the estimate.

**Low:** Further research is very likely to have an important impact on our confidence in the estimate of effect and is likely to change the estimate.

**Very low**: Any estimate of effect is very uncertain

**eTable 3-E** Summary of findings for AEs: GRADE of direct and indirect comparisons of monoclonal antibodies

| **Certainty assessment** | | | | | | | **№ of patients** | | **Effect** | | **Certainty** | **Importance** |
| --- | --- | --- | --- | --- | --- | --- | --- | --- | --- | --- | --- | --- |
| **№ of studies** | **Study design** | **Risk of bias** | **Inconsistency** | **Indirectness** | **Imprecision** | **Other considerations** | **Biologics** | **placebo** | **Relative (95% CI)** | **Absolute (95% CI)** |  |  |
| **Omalizumab vs Placebo** | | | | | | | | | | | | |
| 4 | randomised trials | not serious | not serious | not serious | not serious | none | 82/84 (97.6%) | 84/146 (57.5%) | **RR 0.85** (0.69 to 1.02) | **9 fewer per 100** (from 18 fewer to 1 more) | ⨁⨁⨁⨁ High | IMPORTANT |
| **Dupilumab vs Placebo** | | | | | | | | | | | | |
| 3 | randomised trials | not serious | serious^a^ | not serious | not serious | none | 382/470 (81.3%) | 256/312 (82.1%) | **RR 0.98** (0.92 to 1.04) | **16 fewer per 1,000** (from 66 fewer to 33 more) | ⨁⨁⨁◯ Moderate^a^ | IMPORTANT |
| **Mepolizumab vs Placebo** | | | | | | | | | | | | |
| 3 | randomised trials | not serious | not serious | not serious | not serious | none | 222/279 (79.6%) | 213/263 (81.0%) | **RR 0.98** (0.91 to 1.07) | **16 fewer per 1,000** (from 73 fewer to 57 more) | ⨁⨁⨁⨁ High | IMPORTANT |
| **Tezepelumab vs Placebo** | | | | | | | | | | | | |
| 1 | randomised trials | not serious | not serious | not serious | not serious | none | 159/203 (78.3%) | 158/205 (77.1%) | **RR 1.02** (0.91 to 1.13) | **15 more per 1,000** (from 69 fewer to 100 more) | ⨁⨁⨁⨁ High | IMPORTANT |
| **Dupilumab vs Mepolizumab** | | | | | | | | | | | | |
| 0 | randomised trials | not serious | serious^b^ | not serious | not serious | none | - | - | **RR 0.99** (0.90 to 1.10) | **1 fewer per 1,000** (from 1 fewer to 1 fewer) | ⨁⨁⨁◯ Moderate^b^ | IMPORTANT |
| **Dupilumab vs Omalizumab** | | | | | | | | | | | | |
| 0 | randomised trials | not serious | serious^b^ | not serious | not serious | none | - | - | **RR 1.15** (0.94 to 1.43) | **1 fewer per 1,000** (from 1 fewer to 1 fewer) | ⨁⨁⨁◯ Moderate^b^ | IMPORTANT |
| **Dupilumab vs Tezepelumab** | | | | | | | | | | | | |
| 0 | randomised trials | not serious | serious^b^ | not serious | not serious | none | - | - | **RR 0.96** (0.85 to 1.09) | **1 fewer per 1,000** (from 1 fewer to 1 fewer) | ⨁⨁⨁◯ Moderate^b^ | IMPORTANT |
| **Mepolizumab vs Omalizumab** | | | | | | | | | | | | |
| 0 | randomised trials | not serious | not serious | not serious | not serious | none | - | - | **RR 1.16** (0.94 to 1.45) | **1 fewer per 1,000** (from 1 fewer to 1 fewer) | ⨁⨁⨁⨁ High | IMPORTANT |
| **Mepolizumab vs Tezepelumab** | | | | | | | | | | | | |
| 0 | randomised trials | not serious | not serious | not serious | not serious | none | - | - | **RR 0.97** (0.85 to 1.10) | **1 fewer per 1,000** (from 1 fewer to 1 fewer) | ⨁⨁⨁⨁ High | IMPORTANT |
| **Omalizumab vs Tezepelumab** | | | | | | | | | | | | |
| 0 | randomised trials | not serious | not serious | not serious | not serious | none | - | - | **RR 0.83** (0.66 to 1.03) | **1 fewer per 1,000** (from 1 fewer to 1 fewer) | ⨁⨁⨁⨁ High | IMPORTANT |

**CI:** confidence interval; **RR:** risk ratio

**Explanations**

a. Direct evidence downgraded -1 due to heterogeneity.

b. Indirect evidence downgraded -1 due to heterogeneity.

**Grade Definition**

**High:** Further research is very unlikely to change our confidence in the estimate of effect.

**Moderate:** Further research is likely to have an important impact on our confidence in the estimate of effect and may change the estimate.

**Low:** Further research is very likely to have an important impact on our confidence in the estimate of effect and is likely to change the estimate.

**Very low**: Any estimate of effect is very uncertain

**eTable 4**. WMD for NPS, SNOT-22, UPSIT, and NCS and RR for AEs pairwise meta-analysis

| Comparison | | Including | Pair-wise meta-analysis | | I^2^, % |
| --- | --- | --- | --- | --- | --- |
|  |  | RCTs, n | WMD or RR (95% CI) | p value |  |
| **NPS** |  |  |  |  |  |
| Omalizumab | Placebo | 4 | −1.25 (−1.52, −0.97) | <0.00001* | 91 |
| Dupilumab | Placebo | 3 | −2.16 (−2.44, −1.89) | <0.00001* | 30 |
| Mepolizumab | Placebo | 3 | − 0.90 (−1.19, −0.62) | <0.00001* | 0 |
| Tezepelumab | Placebo | 1 | −1.50 (−1.81, −1.19) | <0.00001* | - ^#^ |
| **SNOT-22** |  |  |  |  |  |
| Omalizumab | Placebo | 2 | −15.62 (−19.79, −11.45) | <0.00001* | 0 |
| Dupilumab | Placebo | 5 | −17.87 (−20.34, −15.39) | <0.00001* | 70 |
| Mepolizumab | Placebo | 2 | −13.47(−17.94, −8.99) | <0.00001* | 0 |
| Tezepelumab | Placebo | 1 | −13.50(−17.31, −9.69) | <0.00001* | - ^#^ |
| **UPSIT** |  |  |  |  |  |
| Omalizumab | Placebo | 2 | 3.84（2.19，5.50） | <0.00001* | 0 |
| Dupilumab | Placebo | 3 | 10.95 (9.73, 12.16) | <0.00001* | 51 |
| Mepolizumab | Placebo | 1 | 1.30 (−0.59, 3.19) | 0.18 | - ^#^ |
| Tezepeluab | Placebo | 1 | 6.70 (5.21, 8.19) | <0.00001* | - ^#^ |
| **NCS**^&^ |  |  |  |  |  |
| Omalizumab | Placebo | 3 | −0.67 (−0.87, −0.48) | <0.00001* | 83 |
| Dupilumab | Placebo | 3 | −0.90 (−1.04, −0.77) | <0.00001* | 0 |
| Tezepeluab | Placebo | 1 | −0.64 (−0.78, −0.50) | <0.00001* | - ^#^ |
| **AEs** |  |  |  |  |  |
| Omalizumab | Placebo | 4 | 0.88 (0.73, 1.07) | 0.21 | 0 |
| Dupilumab | Placebo | 3 | 0.96 (0.90, 1.03) | 0.23 | 71 |
| Mepolizumab | Placebo | 3 | 0.99 (0.91, 1.08) | 0.84 | 28 |
| Tezepelumab | Placebo | 1 | 1.02 (0.92,1.13) | 0.76 | - ^#^ |
| RR (95% CI) in blue color. *, p < 0.05; ^#^, not compared. ^&^, in mepolizumab, nasal blockage was assessed by the visual analog scale score (not NCS) so that there were no data for the meta-analysis. WMD, weighted mean difference; RR, risk ratio; CI, confidence interval. | | | | | |
|  |  |  |  |  |  |
|  |  |  |  |  |  |
|  |  |  |  |  |  |

**eTable 5**. WMD for NPS, SNOT-22, UPSIT, and NCS and RR for AEs NMA

| Outcomes | | WMD or RR (95% CI) | | | | |
| --- | --- | --- | --- | --- | --- | --- |
| **NPS** |  | |  |  |  |  |
|  | Dupilumab | |  |  |  |  |
|  | -1.26 (-1.65, -0.86) * | | Mepolizumab |  |  |  |
|  | -0.92 (-1.31, -0.53) * | | 0.34 (-0.05, 0.73) | Omalizumab |  |  |
|  | -0.67 (-1.08, -0.25) * | | 0.59 (0.17, 1.01) * | 0.25 (-0.16, 0.67) | Tezepelumab |  |
|  | -2.16 (-2.44, -1.89) * | | -0.90 (-1.18, -0.62) * | -1.25 (-1.51, -0.97) * | -1.50 (-1.81, -1.19) * | Placebo |
| **SNOT-22** |  | |  |  |  |  |
|  | Dupilumab | |  |  |  |  |
|  | -4.40 (-9.54, 0.76) | | Mepolizumab |  |  |  |
|  | -2.24 (-7.09, 2.64) | | 2.18 (-3.97, 8.29) | Omalizumab |  |  |
|  | -4.35 (-8.89, 0.17) | | 0.033 (-5.87, 5.96) | -2.12 (-7.77, 3.53) | Tezepelumab |  |
|  | -17.86 (-20.33, -15.39) * | | -13.47(-17.97, -8.99) * | -15.63 (-19.83, -11.48) * | -13.51 (-17.32, -9.72) * | Placebo |
| **UPSIT** |  | |  |  |  |  |
|  | Dupilumab | |  |  |  |  |
|  | 9.19 (6.93, 11.45) * | | Mepolizumab |  |  |  |
|  | 6.63 (4.57, 8.69) * | | -2.56 (-5.06, -0.05) * | Omalizumab |  |  |
|  | 3.78(1.85, 5.71) * | | -5.42 (-7.83, -2.99)* | -2.85 (-5.08, -0.63) * | Tezepelumab |  |
|  | 10.48 (9.23, 11.70) * | | 1.28 (-0.60, 3.16) | 3.84 (2.19, 5.50) * | 6.70 (5.21, 8.18) * | Placebo |
| **NCS** |  | |  |  |  |  |
|  | Dupilumab | |  |  |  |  |
|  | - | | Mepolizumab |  |  |  |
|  | -0.23 (-0.46, 0.002) | | - | Omalizumab |  |  |
|  | -0.26 (-0.45, -0.069) * | | - | -0.03 (-0.27, 0.20) | Tezepelumab |  |
|  | -0.90 (-1.04, -0.77) * | | - | -0.67 (-0.86, -0.48) * | -0.65 (-0.78, -0.50) * | Placebo |
| **AEs** |  | |  |  |  |  |
|  | Dupilumab | |  |  |  |  |
|  | 0.99 (0.9, 1.1) | | Mepolizumab |  |  |  |
|  | 1.15 (0.94, 1.43) | | 1.16 (0.94, 1.45) | Omalizumab |  |  |
|  | 0.96 (0.85, 1.09) | | 0.97 (0.85, 1.1) | 0.83 (0.66, 1.03) | Tezepelumab |  |
|  | 0.98 (0.92, 1.04) | | 0.98 (0.91, 1.07) | 0.85 (0.69, 1.02) | 1.02 (0.91, 1.13) | Placebo |
| RR (95% CI) in blue color; *, p < 0.05; WMD, weighted mean difference; RR, risk ratio; CI, confidence interval; NPS, nasal polyp score; SNOT-22, Sino-Nasal Outcome Test-22; UPSIT, University of Pennsylvania Smell Identification Test; NCS, nasal congestion score; AEs, adverse events; NMA, network meta-analysis. | | | | | | |

**eTable 6**. SUCRA, PrBest and mean rank of all outcomes

| Outcomes | | Placebo | Intervention | | | | |
| --- | --- | --- | --- | --- | --- | --- | --- |
|  |  |  | Omalizumab | Dupilumab | Mepolizumab |  | Tezepelumab |
| **NPS** | SUCRA | 0.000 | 0.518 | 0.999 | 0.262 | 0.720 | |
|  | PrBest | 0 | 0 | 0.999 | 0 | 0.001 | |
|  | Mean rank | 4.9 | 2.9 | 1.0 | 3.9 | 2.1 | |
| **SNOT-22** | SUCRA | 0 | 0.677 | 0.935 | 0.447 | 0.442 | |
|  | PrBest | 0 | 0.176 | 0.768 | 0.036 | 0.021 | |
|  | Mean rank | 5.0 | 2.3 | 1.2 | 3.21 | 3.23 | |
| **UPSIT** | SUCRA | 0.023 | 0.496 | 0.999 | 0.233 | 0.749 | |
|  | PrBest | 0 | 0 | 0.999 | 0 | 0 | |
|  | Mean rank | 4.9 | 3.0 | 1.0 | 4.0 | 2.0 | |
| **NCS** | SUCRA | 0.000 | 0.545 | 0.990 | -^*^ | 0.465 | |
|  | PrBest | 0 | 0.026 | 0.971 | -^*^ | 0.003 | |
|  | Mean rank | 4.0 | 2.3 | 1.0 | -^*^ | 2.6 | |
| **AEs** | SUCRA | 0.694 | 0.064 | 0.461 | 0.532 | 0.749 | |
|  | PrBest | 0.188 | 0.023 | 0.094 | 0.175 | 0.52 | |
|  | Mean rank | 2.2 | 4.7 | 3.2 | 2.9 | 2.0 | |

^*^, not compared. SUCRA, surface under the cumulative ranking curve; NPS, nasal polyp score; SNOT-22, Sino-Nasal Outcome Test-22; UPSIT, University of Pennsylvania Smell Identification Test; NCS, nasal congestion score; AEs, adverse events.

**eTable 7**. Risk of bias of RCTs

| **RCTs** | **Intervention** | **Randomization process** | **Deviation from intended intervention** | **Missing outcome data** | **Measurement of outcome** | **Selection of overall result** | **Overall bias** |
| --- | --- | --- | --- | --- | --- | --- | --- |
| **NPS** |  |  |  |  |  |  |  |
| Bachert2019a  (SINUS 24) ^[12]^ | Dupilumab | Low | Low | Low | Low | Low | Low |
| Bachert2019b  (SINUS 52) ^[12]^ | Dupilumab | Low | Low | Low | Low | Low | Low |
| Bachert2016^[11]^ | Dupilumab | Low | Low | Low | Low | Low | Low |
| Han2021  (SYNAPSE) ^[9]^ | Mepolizumab | Low | Low | Low | Low | Low | Low |
| Bachert2017^[8]^ | Mepolizumab | Low | Low | Low | Low | Low | Low |
| Gevaert2011^[10]^ | Mepolizumab | Low | Low | Some | Low | Low | Some |
| Gevaert2020a  (POLYP 1) ^[6]^ | Omalizumab | Low | Low | Low | Low | Low | Low |
| Gevaert2020b  (POLYP 2) ^[6]^ | Omalizumab | Low | Low | Low | Low | Low | Low |
| Gevaert2013^[42]^ | Omalizumab | Low | Low | Low | Some | Low | Some |
| Pinto2010^[7]^ | Omalizumab | Low | Low | Low | Some | Low | Some |
| Lipworth2025^[23]^ | Tezepelumab | Low | Low | Low | Low | Low | Low |
| **SNOT-22** |  |  |  |  |  |  |  |
| Bachert2019a  (SINUS 24) ^[12]^ | Dupilumab | Low | Low | Low | Some | Low | Some |
| Bachert2019b  (SINUS 52) ^[12]^ | Dupilumab | Low | Low | Low | Some | Low | Some |
| Bachert2016^[11]^ | Dupilumab | Low | Low | Low | Some | Low | Some |
| LIBERTY  QUEST | Dupilumab | Low | Low | Low | Low | Low | Low |
| LIBERTY VENTURE | Dupilumab | Low | Low | Low | Low | Low | Low |
| Han2021  (SYNAPSE) ^[9]^ | Mepolizumab | Low | Low | Low | Some | Low | Some |
| Bachert2017^[8]^ | Mepolizumab | Low | Low | Low | Some | Low | Some |
| Gevaert2020a  (POLYP 1) ^[6]^ | Omalizumab | Low | Low | Low | Low | Low | Low |
| Gevaert2020b  (POLYP 2) ^[6]^ | Omalizumab | Low | Low | Low | Low | Low | Low |
| Lipworth2025^[23]^ | Tezepelumab | Low | Low | Low | Some | Low | Some |
| **UPSIT** |  |  |  |  |  |  |  |
| Bachert2019a  (SINUS 24) ^[12]^ | Dupilumab | Low | Low | Low | Some | Low | Some |
| Bachert2019b  (SINUS 52) ^[12]^ | Dupilumab | Low | Low | Low | Some | Low | Some |
| Bachert2016^[11]^ | Dupilumab | Low | Low | Low | Some | Low | Some |
| Han2021  (SYNAPSE) ^[9]^ | Mepolizumab | Low | Low | Low | Some | Low | Some |
| Gevaert2020a  (POLYP 1) ^[6]^ | Omalizumab | Low | Low | Low | Low | Low | Low |
| Gevaert2020b  (POLYP 2) ^[6]^ | Omalizumab | Low | Low | Low | Low | Low | Low |
| Lipworth2025^[23]^ | Tezepelumab | Low | Low | Low | Low | Low | Low |
| **NCS** |  |  |  |  |  |  |  |
| Bachert2019a  (SINUS 24) ^[12]^ | Dupilumab | Low | Low | Low | Low | Low | Low |
| Bachert2019b  (SINUS 52) ^[12]^ | Dupilumab | Low | Low | Low | Low | Low | Low |
| Bachert2016 | Dupilumab | Low | Low | Low | Some | Low | Some |
| Gevaert2020a  (POLYP 1) ^[6]^ | Omalizumab | Low | Low | Low | Low | Low | Low |
| Gevaert2020b  (POLYP 2) ^[6]^ | Omalizumab | Low | Low | Low | Low | Low | Low |
| Gevaert2013^[42]^ | Omalizumab | Low | Low | Low | Some | Low | Some |
| Lipworth2025^[23]^ | Tezepelumab | Low | Low | Low | Low | Low | Low |
| **AEs** |  |  |  |  |  |  |  |
| Bachert2019a  (SINUS 24) ^[12]^ | Dupilumab | Low | Low | Low | Low | Low | Low |
| Bachert2019b  (SINUS 52) ^[12]^ | Dupilumab | Low | Low | Low | Low | Low | Low |
| Bachert2016^[11]^ | Dupilumab | Low | Low | Low | Low | Low | Low |
| Han2021  (SYNAPSE) ^[9]^ | Mepolizumab | Low | Low | Low | Low | Low | Low |
| Bachert2017^[8]^ | Mepolizumab | Low | Low | Low | Low | Low | Low |
| Gevaert2011^[10]^ | Mepolizumab | Low | Low | Low | Low | Low | Low |
| Gevaert2020a  (POLYP 1) ^[6]^ | Omalizumab | Low | Low | Low | Low | Low | Low |
| Gevaert2020b  (POLYP 2) ^[6]^ | Omalizumab | Low | Low | Low | Low | Low | Low |
| Gevaert2013^[42]^ | Omalizumab | Low | Low | Low | Low | Low | Low |
| Pinto2010^[7]^ | Omalizumab | Low | Low | Low | Low | Low | Low |
| Lipworth2025^[23]^ | Tezepelumab | Low | Low | Low | Low | Low | Low |
|  |  |  |  |  |  |  |  |

All studies were placebo controlled. RCTs, randomized controlled trials. NPS, nasal polyp score; SNOT-22, Sino-Nasal Outcome Test-22; UPSIT, University of Pennsylvania Smell Identification Test; NCS, nasal congestion score; AEs, adverse events; NMA, network meta-analysis.

**eTable 8**. Sensitivity analyses of efficacy outcomes of biologics versus placebo

| **Outcomes** | **Primary analysis** | **Excluding some concerns bias** | | | **Excluding <24-week trials** |
| --- | --- | --- | --- | --- | --- |
| **NPS** |  | |  | |  |
| Omalizumab | −1.25 (−1.52, −0.97) | | -0.87 (-1.19, -0.55) | | -0.83 (-1.14, -0.51) |
| Dupilumab | −2.16 (−2.44, −1.89) | | -2.16 (-2.44, -1.89) | | -2.22 (-2.51, -1.93) |
| Mepolizumab | − 0.90 (−1.19, −0.62) | | -0.87 (-1.16, -0.57) | | -0.87 (-1.16, -0.57) |
| Tezepelumab | −1.50 (−1.81, −1.19) | | -1.50 (-1.81, -1.19) | | -1.50 (-1.81, -1.19) |
| **SNOT-22** |  | |  | |  |
| Omalizumab | −15.62 (−19.79, −11.45) | | -15.62 (-19.79, -11.45) | | -15.62 (-19.79, -11.45) |
| Dupilumab | −17.87 (−20.34, −15.39) | | -10.60 (-15.31, -5.89) | | -17.84 (-20.45, -15.23) |
| Mepolizumab | −13.47(−17.94, −8.99) | | Not estimable | | -13.47 (-17.94, -8.99) |
| Tezepelumab | −13.50(−17.31, −9.69) | | Not estimable | | -13.50 (-17.31, -9.69) |
| **UPSIT** |  | |  | |  |
| Omalizumab  Dupilumab  Mepolizumab  Tezepelumab | 3.84（2.19，5.50）  10.96 (9.72, 12.19)  1.30 (−0.59, 3.19)  6.70 (5.21, 8.19) | | 3.84 (2.19, 5.50)  14.80 (10.86, 18.74)  Not estimable  6.70 (5.21, 8.19) | | 3.84 (2.19, 5.50)  10.54 (9.27, 11.82)  1.30 (-0.59, 3.19)  6.70 (5.21, 8.19) |
| **NCS** |  | |  | |  |
| Omalizumab  Dupilumab  Tezepelumab | −0.67 (−0.87, −0.48)  −0.90 (−1.04, −0.77)  −0.64 (−0.78, −0.50) | | -0.52 (-0.73, -0.31)  -0.93 (-1.07, -0.79)  -0.64 (-0.78, -0.50) | | -0.67 (-0.87, -0.48)  -0.93 (-1.07, -0.79)  -0.64 (-0.78, -0.50) |
| **AEs** |  | |  | |  |
| Omalizumab | 0.88 (0.73, 1.07) | | 0.88 (0.73, 1.07) | | 0.88 (0.73, 1.07) |
| Dupilumab | 0.96 (0.90, 1.03) | | 0.96 (0.90, 1.03) | | 0.94 (0.88, 1.01) |
| Mepolizumab | 0.99 (0.91, 1.08) | | 0.99 (0.91, 1.08) | | 0.97 (0.90, 1.05) |
| Tezepelumab | 1.02 (0.92,1.13) | | 1.02 (0.92, 1.13) | 1.02 (0.92, 1.13) | |

Not estimable outcomes resulted from single-study networks post-exclusion. NPS, nasal polyp score; SNOT-22, Sino-Nasal Outcome Test-22; UPSIT, University of Pennsylvania Smell Identification Test; NCS, nasal congestion score; AEs, adverse events.

**Figure legends**

eFigure 1. A, ‘Risk of bias’ graph: review authors’ judgements about each risk of bias item presented as percentages across all included studies. B, ‘Risk of bias’ summary: review authors’ judgements about each risk of bias item for each included study.

eFigure 2. A, Funnel plots for NPS (nasal polyp score). B, Funnel plots for SNOT-22 (Sino-Nasal Outcome Test-22). C, Funnel plots for UPSIT (University of Pennsylvania Smell Identification Test). D, Funnel plots for NCS (nasal congestion score). E, Funnel plots for AEs (adverse events).


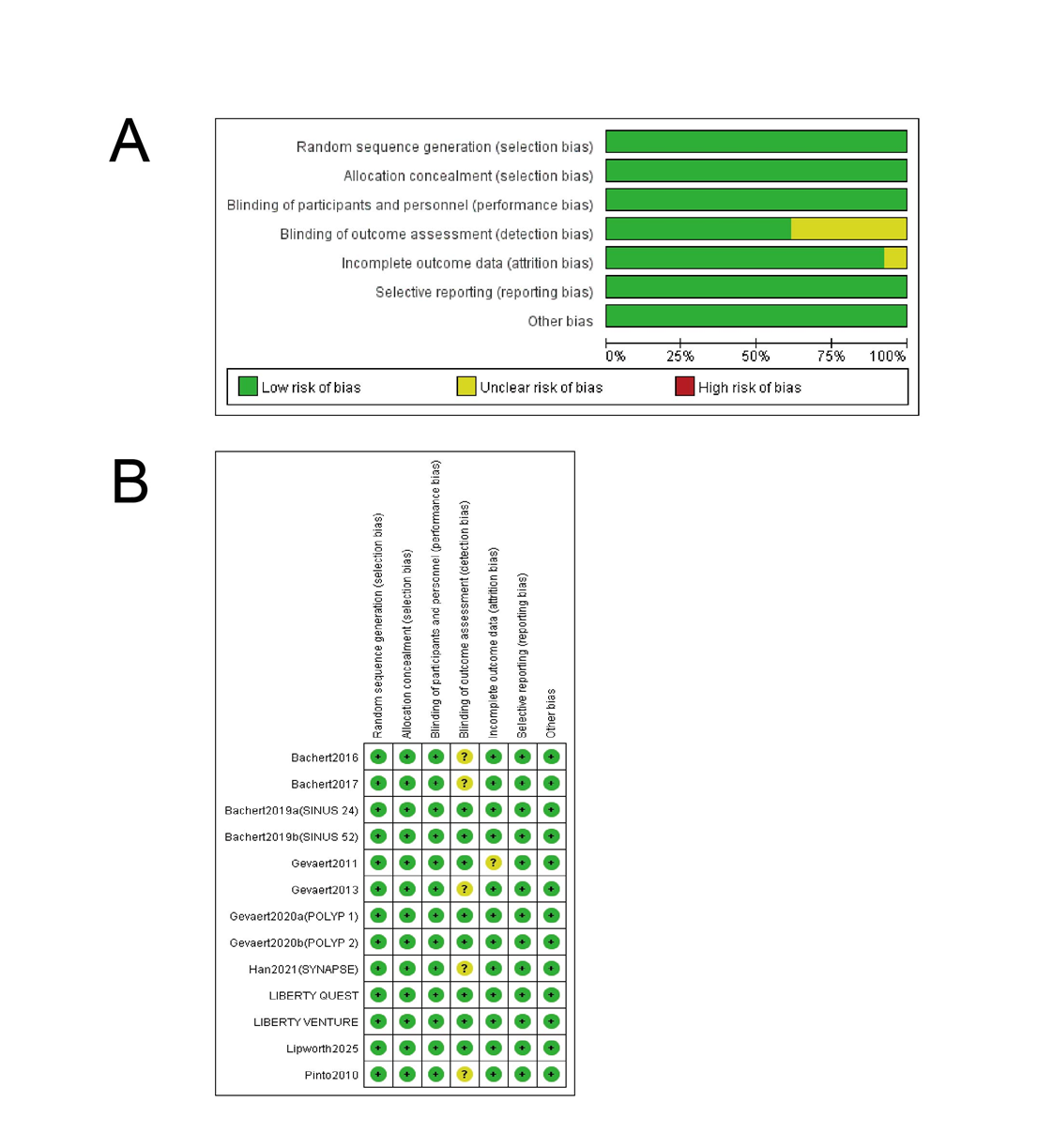


eFigure 1. A, ‘Risk of bias’ graph: review authors’ judgements about each risk of bias item presented as percentages across all included studies. B, ‘Risk of bias’ summary: review authors’ judgements about each risk of bias item for each included study.


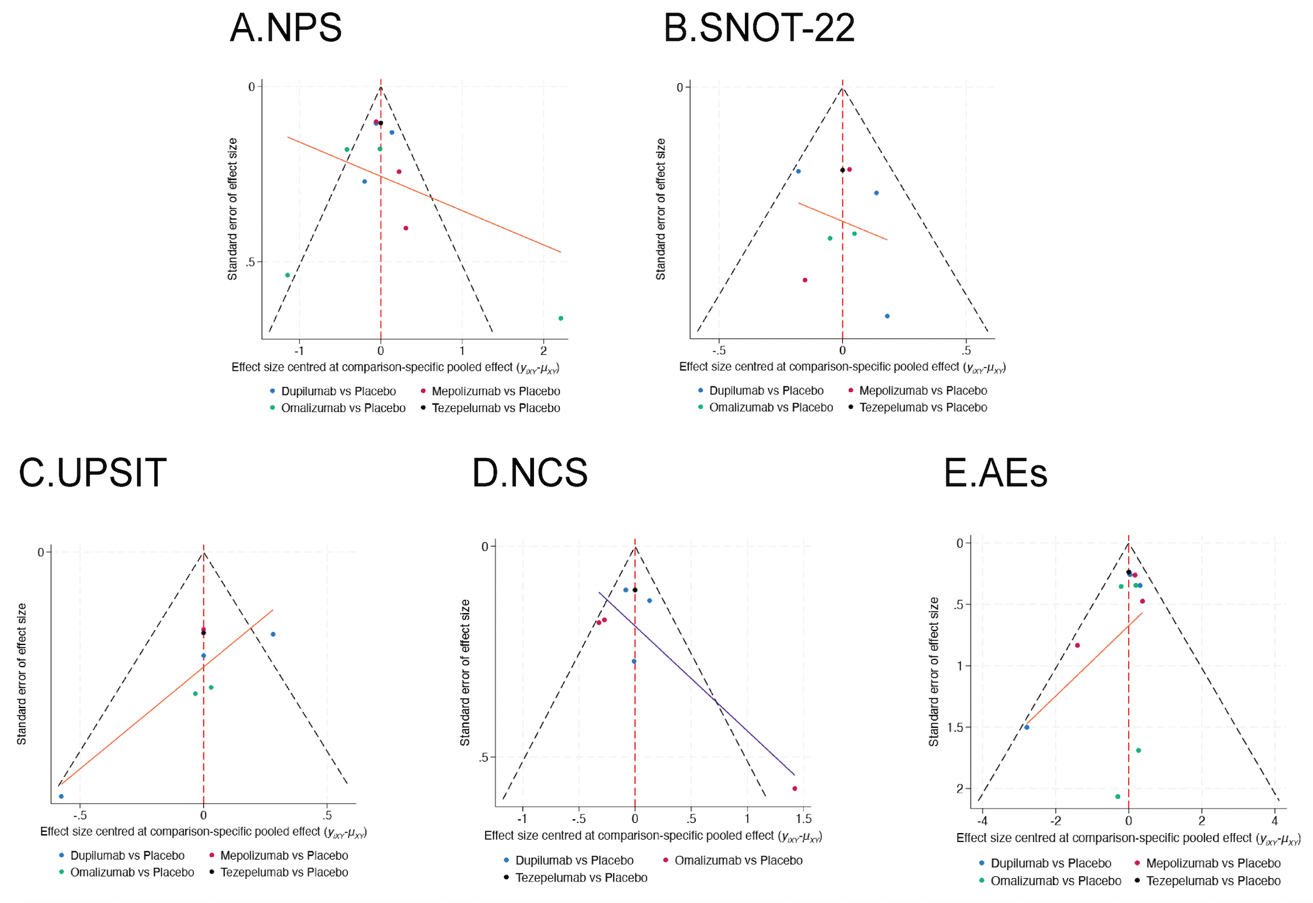


eFigure 2. A, Funnel plots for NPS (nasal polyp score). B, Funnel plots for SNOT-22 (Sino-Nasal Outcome Test-22). C, Funnel plots for UPSIT (University of Pennsylvania Smell Identification Test). D, Funnel plots for NCS (nasal congestion score). E, Funnel plots for AEs (adverse events).
